# Global Synthesis of Drought Effects on Maize and Wheat Production

**DOI:** 10.1371/journal.pone.0156362

**Published:** 2016-05-25

**Authors:** Stefani Daryanto, Lixin Wang, Pierre-André Jacinthe

**Affiliations:** Department of Earth Sciences, Indiana University-Purdue University Indianapolis (IUPUI), Indianapolis, 46202, United States of America; Tennessee State University, UNITED STATES

## Abstract

Drought has been a major cause of agricultural disaster, yet how it affects the vulnerability of maize and wheat production in combination with several co-varying factors (i.e., phenological phases, agro-climatic regions, soil texture) remains unclear. Using a data synthesis approach, this study aims to better characterize the effects of those co-varying factors with drought and to provide critical information on minimizing yield loss. We collected data from peer-reviewed publications between 1980 and 2015 which examined maize and wheat yield responses to drought using field experiments. We performed unweighted analysis using the log response ratio to calculate the bootstrapped confidence limits of yield responses and calculated drought sensitivities with regards to those co-varying factors. Our results showed that yield reduction varied with species, with wheat having lower yield reduction (20.6%) compared to maize (39.3%) at approximately 40% water reduction. Maize was also more sensitive to drought than wheat, particularly during reproductive phase and equally sensitive in the dryland and non-dryland regions. While no yield difference was observed among regions or different soil texture, wheat cultivation in the dryland was more prone to yield loss than in the non-dryland region. Informed by these results, we discuss potential causes and possible approaches that may minimize drought impacts.

## Introduction

Cereals grains have nourished humanity since their domestication thousands of years ago and remained the most important source of calories for the majority of human population. Three major cereal grains (i.e., maize, wheat and rice) and other minor grains (e.g., barley, sorghum, oat, rye, millet) provided about 56% of the food energy and 50% of the protein consumed on earth [1]. Maize and wheat alone contributed to more than 50% of global cereal production in 2013 or equal to about 1,016 and 713 million tons of grain production, respectively (Table 1). These numbers need to be increased by 60% to 110% by 2050 to meet the increasing human population, meat and dairy consumption, as well as biofuel industry [2].

**Table 1:** World production and top producers of different cereal crops [3].

Common name	Scientific name	Production in tons (x10^6^) in 2013	Top producers in descending order, averaged from 1993 to 2013
**Wheat**	*Triticum* spp.	713.2 (25.65%)*	China, India, USA, Russia
Common wheat, bread wheat	*Triticum aestivum*		
Durum wheat	*Triticum durum*		
Spelt	*Triticum spelta*		
**Rice**	Oryza spp.	745.7 (26.82%)	China, India, Indonesia, Bangladesh
Rice, paddy	*Oryza sativa*		
**Barley**	*Hordeum* spp.	144. 7 (5.20%)	Russia, Germany, Canada, France, Ukraine
Four-row barley	*Hordeum vulgare*		
Two-row barley	*Hordeum disticum*		
Six-row barley	*Hordeum hexasticum*		
**Maize**	*Zea mays*	1,016.7 (36.56%)	China, USA, Brazil, Mexico
Corn, Indian corn, mealies	*Zea mays*		
Pop corn	*Zea mays* var. Everta		
**Rye**	*Secale cereale*	16.7 (0.6%)	Russia, Poland, Germany, Belarus, Ukraine
**Oats**	*Avena* spp.	23.8 (0.86%)	Russia, Canada, USA, Poland, Australia
**Oats**	*Avena sativa*		
**Millets**		29.9 (1.07%)	India, Nigeria, China, Niger
Pearl, cattail millet	*Pennisetum glaucum*		
Proso, common, golden millet	*Panicum miliaceum*		
Barnyard, Japanese millet	*Echinocloa frumentacea*		
African millet, finger	*Eleusine coracana*		
Ditch millet, koda	*Paspalum scrobiculatum*		
Foxtail millet	*Setaria italic*		
**Sorghum**	*Sorghum* spp.	61.4 (2.21%)	USA, India, Nigeria, Mexico, Sudan (former)
Common, milo, feterita, kaffir corn	*Sorghum vulgare*		
Guinea corn	*Sorghum guineense*		
Durra, juwar, kaoliang	*Sorghum dura*		
**Buckwheat**	*Fagopyrum esculentum*	2.55 (0.10%)	China, Russia, Ukraine, France
**Quinoa**	*Chenopodium quinoa*	0.10 (0.004%)	Peru, Bolivia, Ecuador
**Fonio**	*Digitaria* spp.	0.58 (0.20%)	Guinea, Nigeria, Mali, Burkina Faso, Côte d’Ivoire
Fonio, findi	*Digitaria exilis*		
Black fonio, hungry rice	*Digitaria iburua*		
**Triticale**	*Triticale hexaploide*	14.6 (0.52%)	China, USA, Turkey, Vietnam
**Cereals nes**		6.4 (0.23%)	Ethiopia, Chad, Austria, Thailand, Kazakhstan
Canagua or coaihua	*Chenopodium pallidiacaule*		
Quihuicha, Inca wheat	*Amaranthus caudatus*		
Wild rice	*Zizania aquatica*		

*number in parentheses are percentage of total cereal production

Our food source heavily depends on cereals, yet their agricultural production is greatly affected by drought [4, 5]. Drought occurs in virtually all climatic regions and drought-induced crop yield loss is considered among the greatest losses in agriculture. During the last few decades, major drought events have been recorded and are projected to intensify in most parts of Asia and beyond, which could make farming exceedingly challenging in some countries [6]. With the exception of United States, major producers of maize (e.g., China, Brazil, France) and wheat (e.g., China, India, Russia) are projected to experience declines in production due to climate variability [6]. This variability was large enough to offset the increases in production resulting from improvement in technology and elevated carbon dioxide concentration, generating declines in global maize and wheat production by 3.8% and 5.5%, respectively [6]. Similar trend was also found in the less developed regions such as South Asia and Southern Africa where malnourished human population has been concentrated [7]. With global climate change and uncertainties in precipitation patterns, food security may become more vulnerable than in the past [8], yet few economically-viable approaches exist to support crop production under drought [9].

Despite ongoing breeding efforts to develop drought-resistant cultivars [10, 11], prolonged droughts in the food-insecure regions may cause famine, epidemics, and deaths, generate water crisis due to drying up of perennial streams, impact agriculture-based livelihood systems, food security and overall economic development [12]. To fully understand the impact of drought on food security, it is necessary to elucidate the environmental variables and agronomic factors that determine the vulnerability of cereal production to drought. As variabilities often accompanied site-specific field experiments, meta-analysis can be used to summarize results from numerous independent experiments on drought [13]. Based on this reasoning, this study aims to better characterize the co-varying effects of several important factors (i.e., cereal species, agroclimatic regions, plant phenological phases, and soil texture), and ultimately be able to use this information to guide agricultural planning and minimize crop loss due to drought. This information may also aid food production modeling by providing information on drought sensitivity under different co-varying conditions which have been known to constrain the models [14]. Our main research questions are: (i) how does drought-induced maize and wheat yield reduction vary with different phenological phases, agroclimatic regions, and soil texture, and (ii) what can we learn from this analysis to minimize maize and wheat yield reduction?

## Methods

Peer-reviewed journal articles published in English from 1980 to 2015 were collected to build the database based on Google Scholar search using the following two sets of keywords: (i) wheat or maize, water, stress, yield, and field, or (ii) wheat or maize, irrigation, deficit, yield, and field. Only articles that meet the following criteria were included in the database: (i) plants that experienced drought under field conditions (excluding pot studies), (ii) the effect of water deficit was considered in comparison with well-watered condition and not in combination with other treatments (e.g., addition of fertilizers or growth hormones, modification of temperature or CO_2_), (iii) the reported plants were monoculture cereals of maize (*Zea mays*) and wheat (i.e., bread wheat; *Triticum aestivum*) which contributed approximately 62.3% of global cereal production (Table 1), (iv) the articles reported crop response as yield (i.e., grain) [15]. The magnitude of yield responses is examined based on the following categorical variables: (i) cereal species (maize and wheat), (ii) agro-ecosystem types (dryland and non-dryland), (iii) drought timing (vegetative phase, reproductive phase which included flowering and grain filling, and during both the vegetative and reproductive phases or throughout season), and (iv) soil texture (fine-, medium-, or coarse-textured soil).

**Fig 1:**
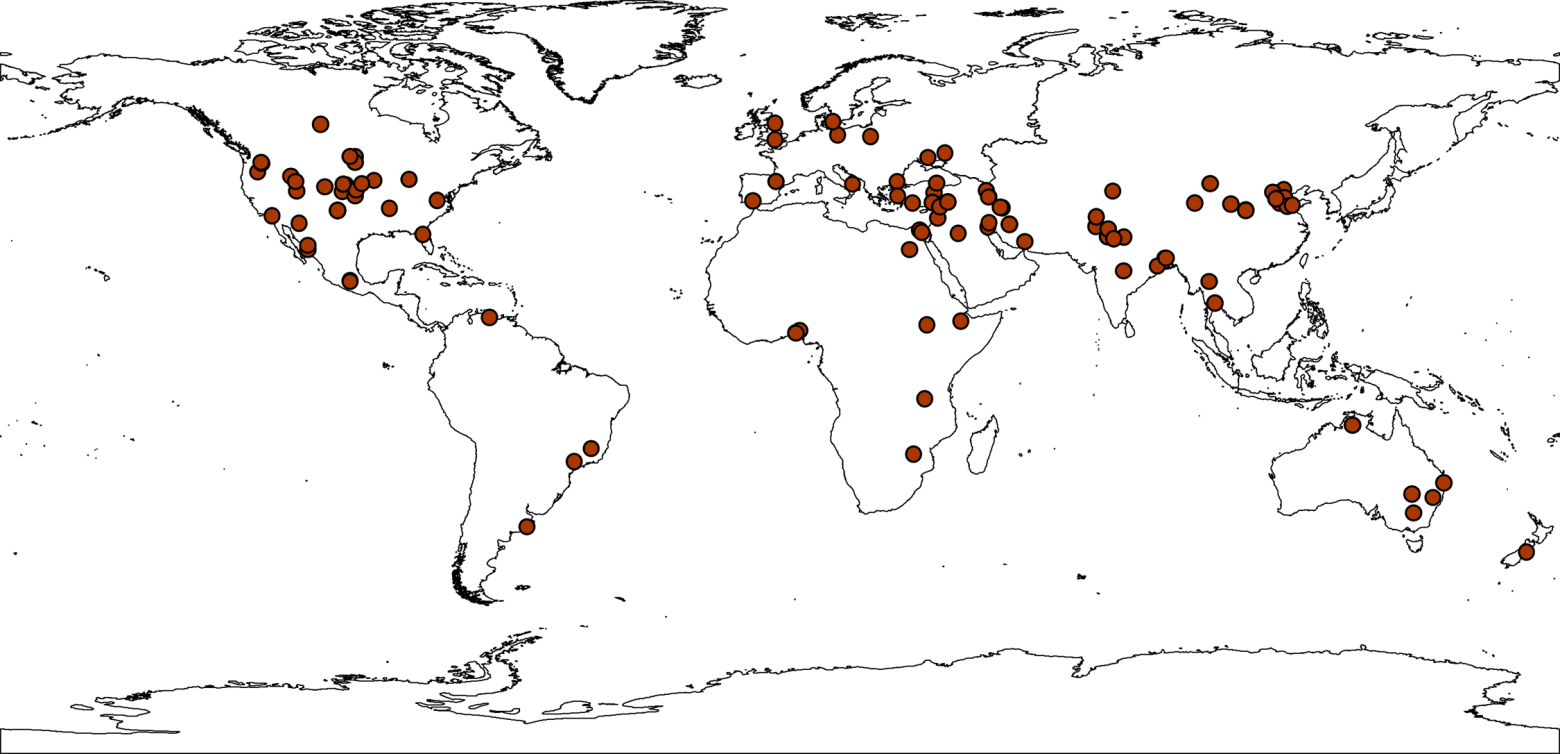
Distribution of the locations of all the studies used in this synthesis. The map was generated using ArcGIS 10.0 (ESRI, Redlands, CA).

For the purposes of meta-analysis, we established discrete levels for the each of the aforementioned variable and coded each observation accordingly. Since we focused our analysis on the amount of water available and yield, we only included studies which examined the single effect of water reduction to minimize the variability of other agronomic factors (e.g., pests, nutrients and diseases) that might affect yield. More importantly, these other factors were usually controlled during water treatment experiments. For the selected articles, we only used paired study sites and therefore considered that other environmental factors (e.g., temperature, light intensity) were the same between control and droughted condition. Agro-ecosystem types were differentiated based on aridity indices, which showed significant correlation with yield [16] and soil texture was differentiated based on soil texture triangle. We considered clay, sandy-clay, and silty-clay soils as fine texture, silt, silt-loam, silty-clay-loam, loam, sandy clay-loam soils, and clay-loam soils as medium texture, and sand, loamy-sand, and sandy-loam as coarse texture [17]. The flowchart diagram on how the process was conducted is presented in S1 Fig. and the PRISMA checklist is available via S1 Information. The distribution of the study locations, generated using ArcGIS 10.0 (ESRI, Redlands, CA), the list of the articles, and raw data extracted from those articles are provided in Fig 1, S2 Information, and S1 Table, respectively.

The total data points before averaging were 2829 from 144 studies. We averaged responses across cultivars under the same drought treatment since the number of cultivars within a species, especially for maize and wheat, could be very large [18–20]. By averaging the response across cultivars instead of considering it as a factor, we increased the reliability of our results and ensured the independence of each data entry [21]. Apart from genetic factors, crop yield was also determined by agronomic practices under different climatic and soil conditions, which were more commonly determined based on species rather than cultivar. We did not differentiate among irrigation types and only recorded the amount of water applied. If a study reported more than one timing of drought or levels of water reduction, all observations were considered independent and included in the database. We therefore established ‘throughout growing season’ as a separate level of drought timing to ensure the independence of data that were continually collected during both vegetative and reproductive phase. Similarly, we also considered all data independent if a study repeated the experiment over several years or locations, except when there was no significant year or location effect. For these non-significant cases, data from the same study were then reported as an average across the years or places to avoid over-representation of the study and to reduce publication bias [21]. Using average for non-significant results, for example, has been reported as a rigorous procedure to ensure the independence of each data entry [21]. After averaging, the total data points used in the meta-analysis were 1164.

Since most of the studies were controlled experiments (i.e., comparing irrigated conditions and irrigation reduction instead of observing natural rainfall deficit), we could not use the widely-accepted drought intensity indices such as Palmer index which are more effective in determining long-term naturally-occurring drought [22, 23]. Instead, we calculated the ratio between water during drought and during well-watered condition (i.e., water availability ratio) for each categorical variable. Since not all studies incorporated the amount of rainfall, we also included or excluded it accordingly (i.e., depending on the study). Therefore, while water availability ratio might or might not include rainfall, the inclusion or exclusion of rainfall was consistent for each ratio. The highest water level in a study (i.e., well-watered condition, ≤100% ET) was then used as control for all data in the corresponding study. We used this observed water availability ratio to: (i) measure differences of available water among different categorical variables using one-way ANOVA, and (ii) define drought sensitivity as the relationship between ratio of yield during drought and during well-watered condition (i.e., yield response ratio) and water availability ratio. We used ratio rather than the actual yield or amount of water to make a more robust comparison among categorical variables since some species might have lower or higher yield potential or water demand than others [24].

Drought sensitivity was also determined for each categorical variable. We separated maize from wheat when calculating drought sensitivity during the reproductive phase and in the non-dryland region since we observed different trends within the group (i.e., having *R*^*2*^ lower than 0.1). Since not all studies recorded the amount of water available, we used the subset of data (the exact numbers of data points were shown in the corresponding figures) that recorded both yield response ratio and water availability ratio to construct the relationship. Confidence interval and prediction band for each drought sensitivity relationship was calculated at the 95% confidence level using Sigmaplot (Systat Software, San Jose, CA). It was noted that yield response ratio and drought sensitivity could be different for the same species due to the different calculation method. The yield response ratio was calculated based on the change of yield under drought conditions vs. control without considering the variability in the amount of water reduction. The difference in sensitivity, on the other hand, was determined by the ratio between yield and water of each species. Therefore, while yield response ratio provided information on the performance of one crop compared to other crops under certain range of water reduction, the results may vary according to the water availability levels. Drought sensitivity will thus provide a more useful indicator for farmers since it shows what will happen in the field if they reduce a certain amount of water. We acknowledge, however, that there are some limitations in the calculation of water sensitivity since it is quite possible that the amounts of water applied to the well-watered control are in excess of crop usage. To minimize the effects of overestimating the water requirement (i.e., maximum yield might have been reached at water supply lower than the observed maximum supply) [25], we did not incorporate water levels higher than the maximum evapotranspiration if this information is provided in the paper.

To compare the differences in yield response ratio between each categorical variable, meta-analysis was used to construct the confidence intervals. In order to include those studies that did not adequately report sample size or standard deviation, we performed an unweighted analysis using the log response ratio (lnR) to calculate bootstrapped confidence limits using the statistical software MetaWin 2.0 [26]. The response ratio is the ratio between the outcome of experimental group (i.e., drought) to that of the control group (i.e., well-watered condition) to estimate the proportional change resulting from experimental drought manipulation. To improve the reliability of lnR in estimating the effect size, we performed a simple diagnostic test using the formula [27]:

xSD(4N321+4N)≥3

where x is the mean, SD is the standard deviation and N is the sample size. Bootstrapping was also iterated 9999 times to improve the probability that the confidence interval was calculated around the cumulative mean effect size for each categorical variable [26]. The sample size of each bootstrapping which reported the amount of water reduction was shown in its corresponding figure. The difference is considered significant if the bootstrap confidence interval did not overlap with each other. A statistical significance level of *P* < 0.05 was used.

## Results and Discussion

### Response of Maize and Wheat to Drought

**Fig 2:**
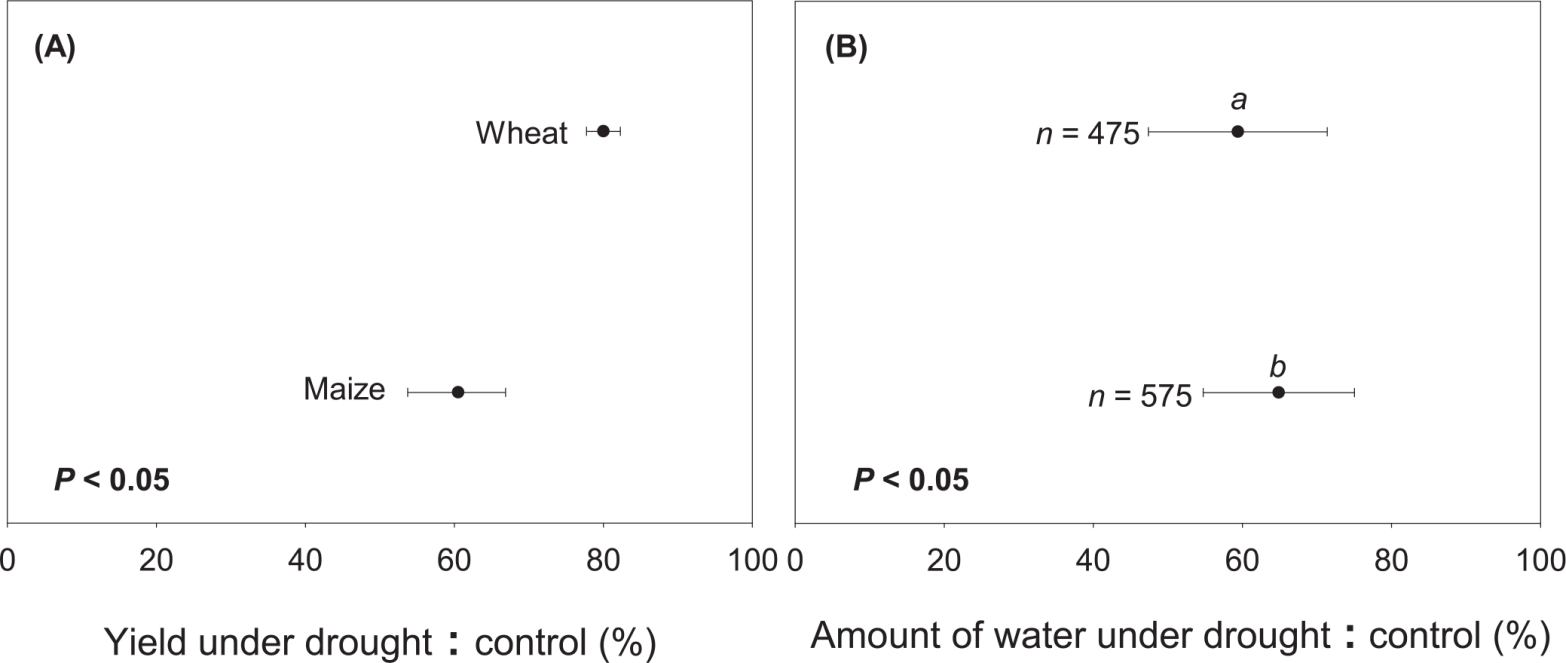
**Observed yield reduction (A) resulting from meta-analysis and their corresponding water reduction (B) for maize and wheat.** Letters *a* and *b* indicate significant difference between observed water reduction level and *n* indicates the number of samples for each category variable that has observable water reduction.

**Fig 3:**
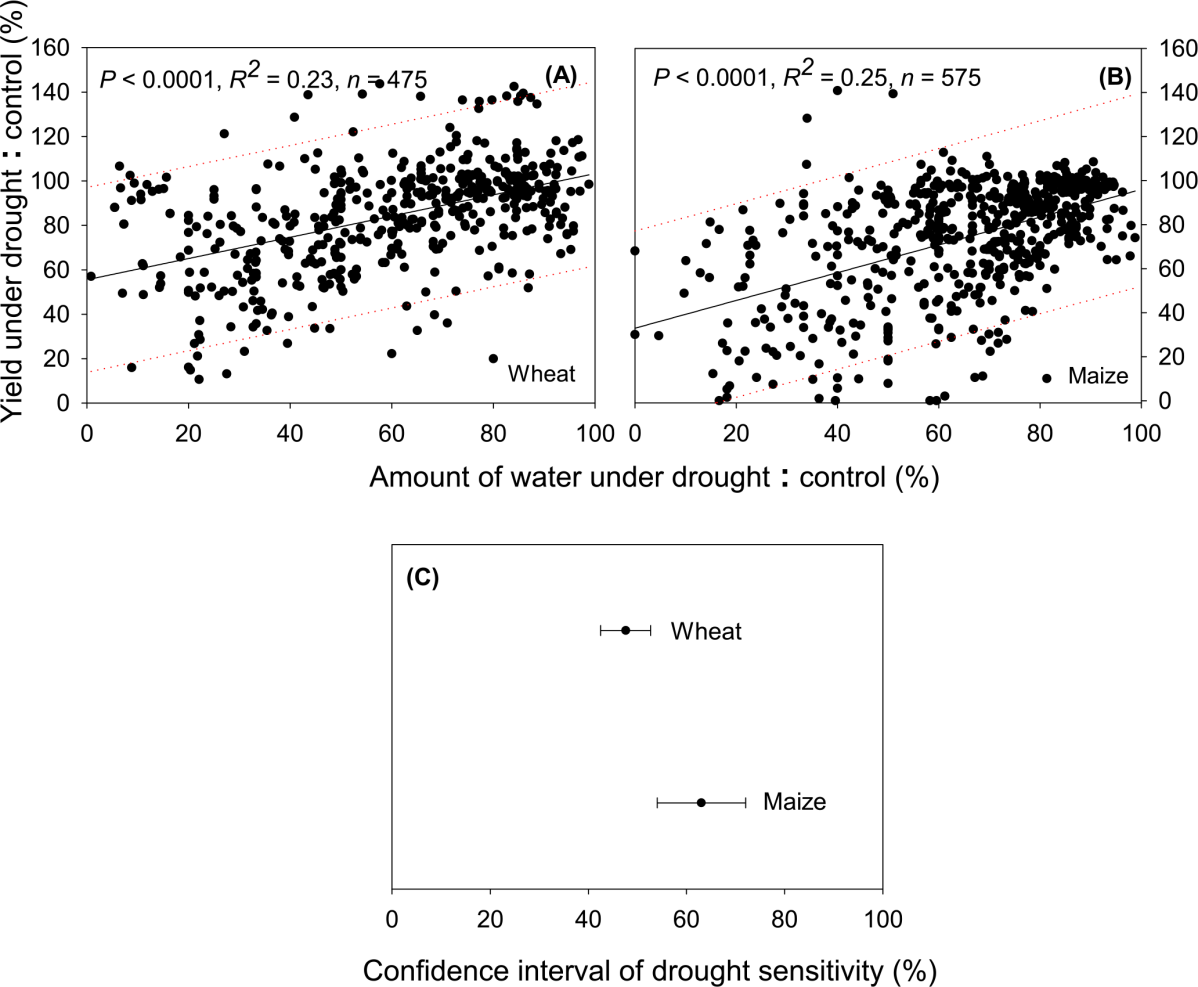
**Drought sensitivity of wheat (A) and maize (B), 95% confidence intervals of drought sensitivity of maize and wheat (C).** Dotted lines indicate 95% prediction band.

Based on the meta-analysis of all the available literature data, our results indicated that maize and wheat showed significantly different yield response to drought. Under comparable water reduction (approximately 40%), wheat had only 20% yield reduction, while maize experienced approximately 39% yield reduction (*P* < 0.05; Fig 2). Our results also indicated that maize, the major C_4_ cereal species (91.7% of global C_4_ cereal production in 2013) had higher sensitivity than wheat, the major C_3_ species (42.9% of global C_3_ cereal production in 2013; Table 1, Fig 3). This result is surprising given that plants with C_4_ photosynthetic pathway usually have higher water-use efficiency than C_3_ plants, and therefore considered less sensitive to drought. Although it has not been studied in all Poaceae family including in wheat and maize, lower chlorophyll contents and higher leaf mortality have been observed in various C_4_ genera (i.e., *Heteropogon*, *Themeda*, *Tristachya*) compared to C_3_ (i.e., *Alloteropsis*, *Panicum*) grasses under the same drought condition (i.e., 48 days without water where soil water content decreased by 0.4% day^-1^) [28]. While photosynthetic rate was significantly higher in those C_4_ than C_3_ grasses under well-watered condition, the opposite occurred when water was limited [29]. Photosynthetic rate of the C_4_ grasses recovered from drought increased more slowly than that of C_3_ grasses with subsequent re-watering [28], which might partly explain the disparity in drought sensitivities between maize and wheat. More importantly, maize is considered a diclinous monoecious plant in which competition for water between female and male flowers during drought favors the development of male rather than female inflorescence [30]. This attribute results in fertilization failure and further contributes to the susceptibility of maize to drought during reproductive phase. In contrast, the same effect is less pronounced in monoclinous monoecious plant (e.g., wheat) as the anthers and ovaries develop in the same flower.

Different drought sensitivities of maize and wheat are likely related to the plant trait variations related to drought. Plant responses to drought are generally divided into three categories: (i) drought escape (e.g., short life cycle), (ii) drought avoidance (i.e., maintenance of favorable water status during drought using various mechanisms such as stomatal closure and senescence of older leaves), and (iii) drought tolerance (i.e., ability of plants to function at low water potential, including the ability to recover after stress) as the result of osmotic adjustment (OA), rigid cell walls, small cells and reactive oxygen species scavenging [31]. Variability of yield response and sensitivity in maize and wheat thus occur as they adopt different adaptation mechanisms to drought. Some plants such as wheat adapt to droughts through their ability to shed their old leaves and maintain appropriate cell turgor and stomatal conductance for carbon assimilation by their young leaves [32, 33]. This stay-green trait (delayed senescence) in wheat is important for its adaptation to environment where late-season drought (i.e., drought during reproductive phase) is a common condition as it allows normal grain filling by mobilizing stem reserve to the grains [34, 35]. Wheat is also able to tolerate drought by having high OA, and along with high transpiration efficiency, they allow recovery from late-season drought, provided that water is available during grain filling period [36]. Osmotic adjustment in wheat facilitates critical growth functions (e.g., root growth, leaf metabolism, pollen development) which may mitigate some of the most detrimental effects of plant water deficit [36]. For maize, strong correlations seem to exist between yield and traits related to reproductive organs due to their sensitivity to drought during reproductive phase [37, 38]. The selection of maize varieties that develop small tassel size has been successful since maize favors the development of male (tassels) rather than female inflorescences (ears), particularly during drought [39]. Small tassel size is positively correlated with yield, which could be due to the suppression of shading and better interception of light by the upper leaves or reduced transpiration [40].

### Different Responses of Plant Phenological Phases to Drought

**Fig 4:**
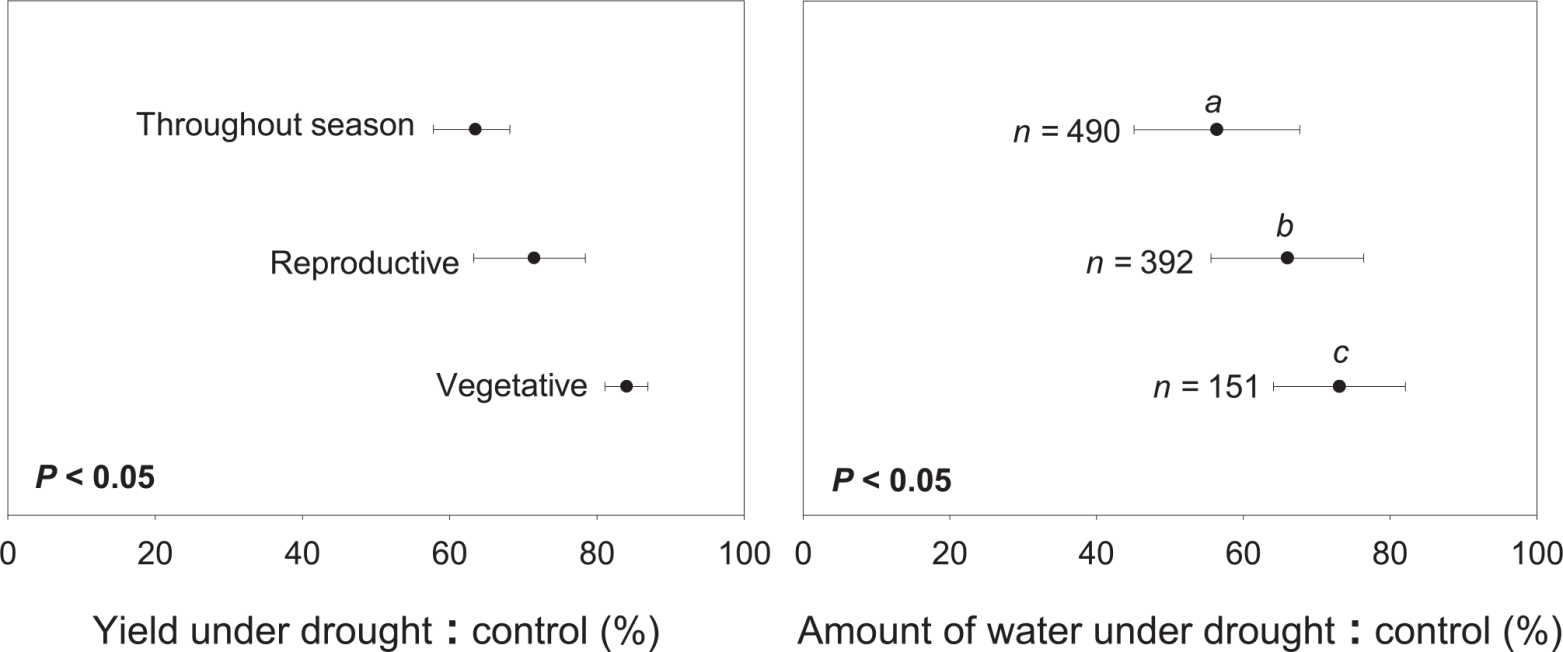
**Observed yield reduction (A) resulting from meta-analysis and their corresponding water reduction (B) for maize and wheat at different phenological phases.** Letters *a*, *b* and *c* indicate significant difference between observed water reduction level and *n* indicates the number of samples for each category that has observable water reduction.

**Fig 5:**
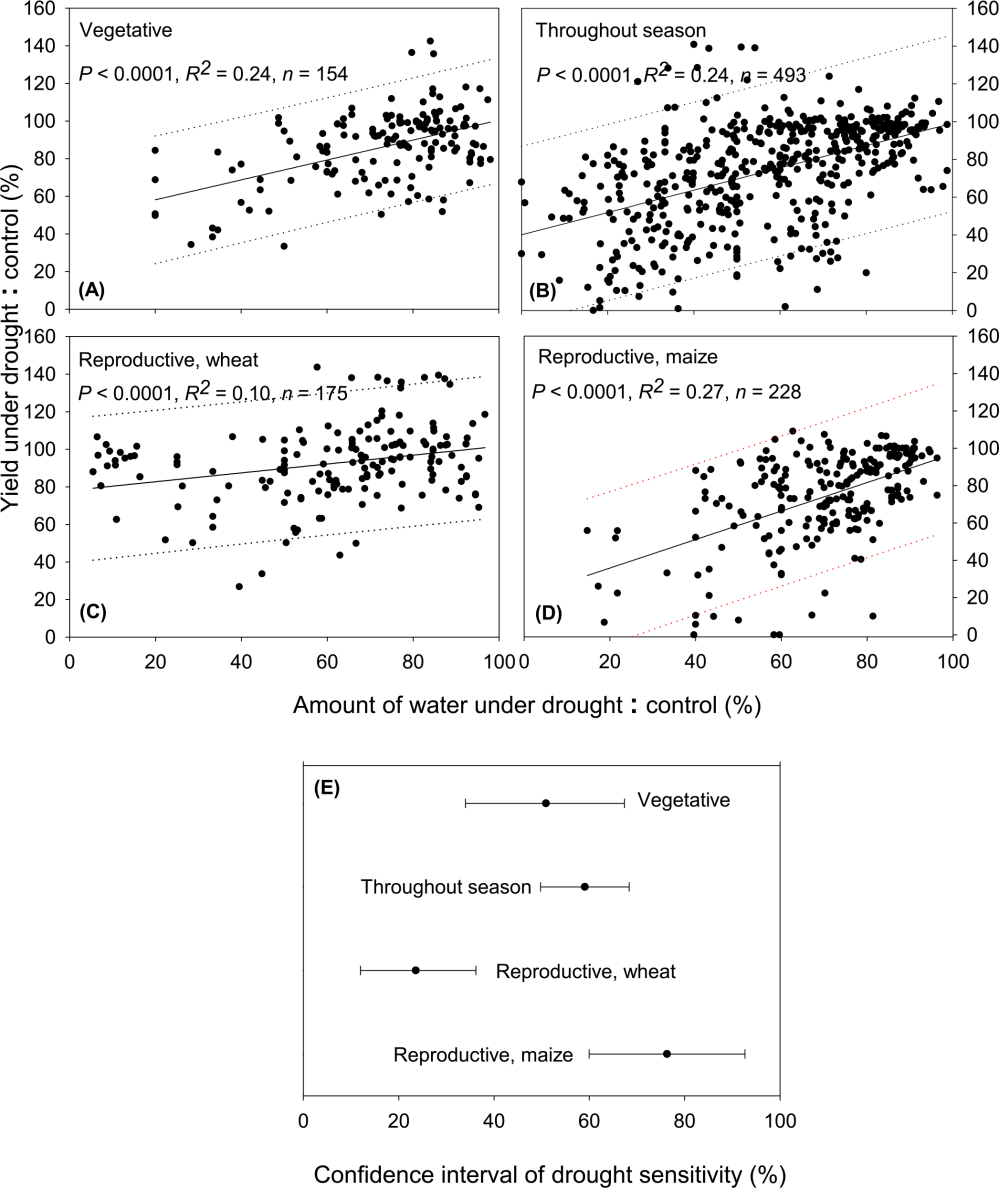
**Drought sensitivity of maize and wheat at different phenological phases (A-D) and 95% confidence interval of drought sensitivity of maize and wheat experiencing drought at different phenological phases (E).** Dotted lines indicate 95% prediction band.

Our results showed that maize and wheat experiencing drought during their reproductive phase had greater yield loss than those experiencing drought during their vegetative phase (*P* < 0.05), although greater amount of available water during vegetative phase likely contributed to the greater yield (Fig 4). While wheat had similar sensitivity to drought during vegetative and reproductive phases, maize was much more sensitive than wheat during the reproductive phase (Fig 5). Plants usually have different sensitivity to drought at different phases of their growing period. Most seed plants, including cereals, start to lose their resistance to drought once they are germinated [41]. In fact, seeding establishment, including crown root and initial green leaf area development is critical. In drought-prone areas, drying seedbed is a common cause of crop failure [41]. This circumstance, however, is not reflected in our analysis since most drought experiments require good germination and initial plant development to observe drought effects during subsequent crop life phases. During plant vegetative phase, we found that yield reduction that occurred due to drought was smaller than that during the reproductive phase (Fig 4), consistent with many other studies [31, 38, 39]. Since most of the carbohydrate in maize and wheat grains is derived from post-anthesis photosynthesis [42], potential yield loss due to drought during vegetative stage is usually small. Some exceptions exist, however, when water stress inhibits ear development and therefore storage capacity [42]. In general, drought during vegetative phase is considered reparable because water deficit merely induced stomatal closure and inhibit photosynthesis which limit carbohydrate synthesis and thus cell division and expansion [31]. Drought that occurred during reproductive phase, however, may lead to ovule abortion and pollen sterility in some cereal species (e.g., rice, maize) [38, 39], suggesting that the effect of drought on yield during this phase could not be reverted by adding water afterwards. Drought causes significant delay in silking [41], preventing the critical synchrony between silking and anthesis and resulting in maize yield reduction [30, 43]. This observation explains why the anthesis-silking interval (ASI) has been reduced in modern maize hybrids [39], and short ASI has been the focus of breeding programs aimed at developing drought-tolerant maize varieties [37, 44]. Indeed, short ASI (and tassel size in temperate maize) have contributed to the increase in grain yield in newer maize hybrids [39].

Wheat seemed to have similar sensitivity to drought, whether it occurred during the vegetative or reproductive phase. This result is surprising given that most of the literature would suggest that the reproductive phase is the most crucial in determining crop yield [31, 33, 38, 39]. This similarity in drought sensitivity between vegetative and reproductive phase might be possible as reduction in the number of ears in wheat was reported when drought occurred during the entire growing season or between anthesis and early milk development, but not when it occurred during grain maturity [45]. Wheat is apparently sensitive to water stress during the vegetative phase (i.e., early-season drought) as it allocates more of its assimilates to the roots, resulting in reduced leaf area, number of leaves per plant, leaf size and leaf longevity [46]. Early-season drought that happened four weeks before anthesis was also found to reduce yield significantly by lowering the number of grains per ear [46, 47]. If drought prolongs to mid-season (i.e., between jointing and anthesis), however, its impact on wheat head size (i.e., the number of spikelet per spike) and root growth can be irreversible since late-emerging tillers do not contribute to yield [48, 49]. Here the combination of reductions in photosynthetic sink and nutrient uptake capacity could severely decrease yield. Therefore, traits that allow soil moisture saving during early growth (e.g., vigorous crop establishment to shade the soil and suppress water competition from weeds, as well as thinner but wider leaves to increase ground cover and radiation-use efficiency) are particularly important for wheat in dry areas where water loss from soil evaporation is significant (e.g., Mediterranean) [50, 51].

### Response to Droughts of Maize and Wheat Grown under Different Climates

Maize and wheat production in various regions of the world is frequently affected by water shortages, particularly in the dry regions. We, however, did not find any significant effect on yield reduction between dryland and non-dryland regions (*P* >0.05; S2 Fig). Wheat plants grown in the dryland had higher sensitivity to drought compared to those grown in the non-dryland region while maize was equally sensitive in both regions (*P* < 0.05; S3 Fig). Drought is one of the most prominent characteristics of the drylands, which makes the two billion inhabitants of these eco-regions vulnerable to crop failure [52]. Given the low relative humidity and the high potential evapotranspiration demand in dryland regions, it is unsurprising that the crops grown there have relatively high sensitivity to drought. Wheat showed lower sensitivity to drought in the non-dryland regions, suggesting that differences in origin and therefore inherited adaptability traits between maize and wheat could attribute to the difference. Unlike maize that likely came from wetter regions [53], wheat originated from dryland region [54], and therefore wheat could show lower sensitivity to drought when they were grown in the more humid regions. Indeed, maize cultivation has been more common in areas where water availability is non-limiting [55]. While 75% of the land area where wheat is grown receives between 375 and 875 mm of annual precipitation with mean temperature of 25°C, wheat can tolerate precipitation ranging between 250 and 1750 mm and temperature from 3° to 32°C [50, 51].

### Response to Drought of Maize and Wheat Grown on Soils of Different Texture

In most cases, soil texture can provide a good estimate for soil-water potential, water holding capacity, and water availability for plant growth. Our meta-analysis results, however, suggested that soil texture was not a major determining factor for drought-induced maize and wheat yield reduction worldwide (S4 Fig). Similarly, soil texture did not affect maize and wheat sensitivity to drought (S5 Fig). Cereal crops, including maize and wheat, usually had larger root mass and root length density when compared to other food crops (i.e., legumes), which showed variability across different soil texture [56, 57]. Cereals also required lower water intake per unit root length compared to legumes [58], and those factors likely contributed to the robustness of cereal yield across contrasting soil textures. Wheat, for example, was able to extend their roots to an average 113 cm and had less than 50% of their total root length in the top 20 cm, while legume (i.e., field pea) to only 65 cm with more than 70% of them was in the top 20 cm [59]. Apart from having drought tolerance strategy (e.g., stay green, high OA), wheat also had drought avoidance strategy by having deep-root system, which allowed them to access subsoil water and performed well under drought [60].

## Conclusions

Overall, we found that maize tended to experience greater yield loss due to drought, partly because maize was originated from wetter region which likely contributed to their sensitivity across agro-climatic conditions. Maize was also highly sensitive during reproductive phase. Although wheat had similar sensitivity to drought during vegetative and reproductive phase, it was considerably lower than that of maize. Therefore, when supplemental irrigation could be applied, the timing of irrigation is essential in determining its efficiency. Our results may be used as the basis to model the interactions between agronomic inputs, to quantify productivity gains and production costs for maize and wheat and to determine optimum irrigation scheduling during critical growth periods (e.g., flowering). Indeed, research in several counties in the Gansu Province of China has shown that supplemental irrigation of wheat and maize using rainwater harvesting stored in subsurface tanks could significantly improve yield in these water-scarce areas (mean annual rainfall = 289 mm). Even a small amount of supplemental irrigation (350–750 m^3^ ha^-1^ or 35–75 mm of water depth equivalent) during the growth period generated about 28–88% increase in yield of wheat and maize, respectively. Thus, the yield increase was more than proportional to the increase (12–25%) in water as supplemental irrigation [9]. Since there is no single approach that is sufficient to improve plant performance, a combination of approaches should be considered when designing a response plan to drought. Site-specific management that considers soil conditions (i.e., intercropping, mulching, and crop rotation) and trait selection that is adjusted to the local climate are more likely to result in sustainable crop yields in a changing climate. Although plant breeding provides a pathway for the development of drought-resistant cereal species and thus could help reduce the vulnerability of agricultural production to the unpredictability of climates, successful applications of these technological innovations require careful consideration of local environmental conditions (e.g., rainfall, temperature and soil nutrients).

## Supporting Information

S1 Fig PRISMA flow diagram of the process of obtaining literature data to build a database for this study. (PDF)

S2 Fig **Ratio between yield under drought and yield under control resulting from meta-analysis (A) and their corresponding water ratio for maize and wheat plants grown in dryland and non-dryland conditions (B).** Letters *a* and *b* indicate significant difference between water under drought and water under control. Letter *n* indicates the number of samples for each category variable that has observable water ratio.
(EPS)

S3 Fig **Drought sensitivity of wheat (A) and maize (B) grown in non-dryland and dryland (C) regions and 95% confidence intervals of drought sensitivity of those plants grown in each of those regions (D).** Dotted lines indicate 95% prediction band.
(EPS)

S4 Fig **Ratio between yield under drought and yield under control resulting from meta-analysis (A) and their corresponding water ratio for maize and wheat plants grown in different soil textures (B).** Letters *a* and *b* indicate significant difference between water under drought and water under control. Letter *n* indicates the number of samples for each category variable that has observable water ratio.
(EPS)

S5 Fig **Drought sensitivity of maize and wheat plants grown in different soil textures (A-C) and 95% confidence intervals of drought sensitivity of cereal crops grown in each of those soils (D).** Dotted lines indicate 95% prediction band.
(EPS)

S1 Information PRISMA checklist of this study. (PDF)

S2 Information List of references used to generate the database for this study. (PDF)

S1 Table Raw data used for generating the results of this study. (PDF)

## References

[1] Cordain L. Cereal grains: Humanity’s double-edged sword In: Simopoulos AP, editor. Evolutionary Aspects of Nutrition and Health Diet, Exercise, Genetics and Chronic Disease. World Review of Nutrition and Dietetics. Basel, Switzerland: Karger; 1999 p. 19–73. 10.1159/000059677 10489816

[2] Ray DK, Mueller ND, West PC, Foley JA. Yield trends are insufficient to double global crop production by 2050. PLOS ONE. 2013 10.1371/journal.pone.0066428 PMC3686737 23840465

[3] FAOSTAT. Available: http://faostat3.fao.org/browse/Q/QC/E. Accessed 19 August 2014.

[4] Kadam NN, Xiao G, Melgar RJ, Bahuguna RN, Quinones C, Tamilselvan A, et al Agronomic and physiological responses to high temperature, drought, and elevated co_2_ interactions in cereals. Advances in Agronomy. 2014;127:111–56.

[5] Elliott J, Deryng D, Müller C, Frieler K, Konzmann M, Gerten D, et al Constraints and potentials of future irrigation water availability on agricultural production under climate change. Proceedings of the National Academy of Sciences. 2014;111(9):3239–44. 10.1073/pnas.1222474110 PMC3948288 24344283

[6] Lobell DB, Schlenker W, Costa-Roberts J. Climate trends and global crop production since 1980. Science. 2011;333(6042):616–20. 10.1126/science.1204531 21551030

[7] Lobell DB, Burke MB, Tebaldi C, Mastrandrea MD, Falcon WP, Naylor RL. Prioritizing climate change adaptation needs for food security in 2030. Science. 2008;319(5863):607–10. 10.1126/science.1152339 18239122

[8] FAO. Climate Change and Food Security: A Framework Document Rome: Food and Agriculture Organization of the United Nations 2008.

[9] Li F, Cook S, Geballe GT, Burch WR Jr. Rainwater Harvesting Agriculture: An Integrated System for Water Management on RainfedLand in China's Semiarid Areas. Ambio. 2000;29(8):477–83.

[10] Bennett DJ, Jennings RC. Successful agricultural innovation in emerging economies: new genetic technologies for global food production: Cambridge University Press; 2013.

[11] Chapman SC, Chakraborty S, Dreccer MF, Howden SM. Plant adaptation to climate change—opportunities and priorities in breeding. Crop and Pasture Science. 2012;63(3):251–68.

[12] Karim MR, Rahman MA. Drought risk management for increased cereal production in Asian least developed countries. Weather and Climate Extremes. 2014. 10.1016/j.wace.2014.10.004.

[13] Hedges LV, Gurevitch J, Curtis PS. The meta-analysis of response ratios in experimental ecology. Ecology. 1999;80(4):1150–6.

[14] Estes LD, Beukes H, Bradley BA, Debats SR, Oppenheimer M, Ruane AC, et al Projected climate impacts to South African maize and wheat production in 2055: a comparison of empirical and mechanistic modeling approaches. Global Change Biology. 2013;19(12):3762–74. 10.1111/gcb.12325 23864352

[15] Daryanto S, Wang L, Jacinthe PA. Global synthesis of drought effects on food legume production. PLOS ONE. 2015;10(6):e0127401 10.1371/journal.pone.0127401 26061704 PMC4464651

[16] Bannayan M, Sanjani S, Alizadeh A, Lotfabadi SS, Mohamadian A. Association between climate indices, aridity index, and rainfed crop yield in northeast of Iran. Field Crops Research. 2010;118(2):105–14.

[17] Soil Texture. Available: ftp://ftp.fao.org/fi/cdrom/fao_training/FAO_Training/General/x6706e/x6706e06.htm. Accessed 8 June 2014.

[18] Louette D, Charrier A, Berthaud J. In situ conservation of maize in Mexico: genetic diversity and maize seed management in a traditional community. Economic Botany. 1997;51(1):20–38.

[19] Stone P, Nicolas M. Wheat cultivars vary widely in their responses of grain yield and quality to short periods of post-anthesis heat stress. Functional Plant Biology. 1994;21(6):887–900.

[20] Hallauer AR, Carena MJ. Maize In: Carena JM, editor. Cereals. New York, NY: Springer US; 2009 p. 3–98.

[21] Lu X, Wang L, McCabe MF. Elevated CO_2_ as a driver of global dryland greening. Scientific reports. 2016;6:20716 10.1038/srep20716 26869389 PMC4751615

[22] Wells N, Goddard S, Hayes MJ. A self-calibrating Palmer drought severity index. Journal of Climate. 2004;17(12):2335–51.

[23] The Climate Data Guide: Palmer Drought Severity Index (PDSI) 2015. Available: https://climatedataguide.ucar.edu/climate-data/palmer-drought-severity-index-pdsi.#sthash.j2i7M8ei.dpuf. Accessed 8 July 2015.

[24] Daryanto S, Wang L, Jacinthe PA. Global synthesis of drought effects on cereal, legume, tuber and root crops production: A review. Agricultural Water Management. 2016 10.1016/j.agwat.2016.04.022

[25] Grassini P, Hall AJ, Mercau JL. Benchmarking sunflower water productivity in semiarid environments. Field Crops Research. 2009;110(3):251–62.

[26] Rosenberg MS, Adams DC, Gurevitch J. MetaWin: Statistical Software for Meta-Analysis Version 2.0 Sunderland, Massachusetts: Sinauer Associates, Inc.; 2000. 133 p.

[27] Lajeunesse M. Bias and correction for the log response ratio in ecological meta-analysis. Ecology. 2015;96(8):2056–63. 26405731 10.1890/14-2402.1

[28] Ripley BS, Frole K, Gilbert ME. Differences in drought sensitivities and photosynthetic limitations between co-occurring C_3_ and C_4_ (NADP-ME) Panicoid grasses. Annals of Botany. 2010;105:493–503. 10.1093/aob/mcp307 20106844 PMC2826257

[29] Ripley BS, Gilbert ME, Ibrahim DG, Osborne CP. Drought constraints on C_4_ photosynthesis: stomatal and metabolic limitations in C_3_ and C_4_ subspecies of *Alloteropsis semialata*. Journal of Experimental Botany. 2007;58:1351–63. 17322550 10.1093/jxb/erl302

[30] Sangoi L, Salvador RJ. Maize susceptibility to drought at flowering: a new approach to overcome the problem. Ciência Rural. 1998;28(4):699–706.

[31] Barnábas B, Jäger K, Fehér A. The effect of drought and heat stress on reproductive processes in cereals. Plant, Cell and Environment. 2008;31:11–38. 17971069 10.1111/j.1365-3040.2007.01727.x

[32] Blum A. Drought resistance, water-use efficiency, and yield potential—are they compatible, dissonant, or mutually exclusive? Australian Journal of Agricultural Research. 2005;56(11):1159–68.

[33] Schaffert RE, Albuquerque PEP, Duarte JO, Garcia JC, Gomide RL, Guimarães CT, et al. Phenotyping sorghum for adaptation to drought. In: Monneveux P, Ribaut JM, editors. Drought phenotyping in crops: from theory to practice CGIAR Generation Challenge Program; 2011. p. 287–99.

[34] Serraj R, Bidinger FR, Chauhan YS, Seetharama N, Nigam SN, Saxena NP. Management of Drought in ICRISAT Cereal and Legume Mandate Crops In: Kijne W, Barker R, Molden D, editors. Water Productivity in Agriculture: Limits and Opportunities for Improvement Wallingford, Oxon, UK: CABI Publishing, CAB International; 2003.

[35] Lopes MS, Araus JL, Van Heerden PD, Foyer CH. Enhancing drought tolerance in C4 crops. Journal of Experimental Botany. 2011;62(9):3135–53. 10.1093/jxb/err105 21511912

[36] Christopher J, Manschadi A, Hammer G, Borrell A. Developmental and physiological traits associated with high yield and stay-green phenotype in wheat. Crop and Pasture Science. 2008;59(4):354–64.

[37] Bolaños J, Edmeades G. The importance of the anthesis-silking interval in breeding for drought tolerance in tropical maize. Field Crops Research. 1996;48(1):65–80.

[38] Fischer KS, Fukai S, Kumar A, Leung H, Jongdee B. Phenotyping rice for adaptation to drought In: Monneveux P, Ribaut JM, editors. Drought phenotyping in crops: from theory to practice: CGIAR Generation Challenge Program; 2011 p. 215–43.

[39] Araus JL, Sanchez C, Edmeades GO. Phenotyping maize for adaptation to drought. In: Monneveux P, Ribaut JM, editors. Drought phenotyping in crops: from theory to practice CGIAR Generation Challenge Program; 2011. p. 263–83.

[40] Monneveux P, Sanchez C, Tiessen A. Future progress in drought tolerance in maize needs new secondary traits and cross combinations. Journal of Agricultural Science. 2008;146:287–300.

[41] Blum A. Crop responses to drought and the interpretation of adaptation Plant Growth Regulation. 1996;20:135–48.

[42] Evans L, Wardlaw I, Fischer R. Wheat In: Evans L, editor. Crop Physiology: Some Case Histories. UK: Cambridge University Press; 1975 p. 101–50.

[43] Edmeades GO, Bolaños J, Elings A, Ribaut JM, Bänziger M, Westgate ME. The Role and Regulation of the Anthesis-Silking Interval in Maize In: Boote MWaK, editor. Physiology and Modeling Kernel Set in Maize. 29: Crop Science Society of America; 2000 p. 43–73.

[44] DuPlessis D, Dijkhuis F. The influence of time lag between pollen shedding and silking on the yield of maize. South African Journal of Agricultural Science. 1967;10:667–74.

[45] Sieling K, Christen O, Richter‐Harder H, Hanus H. Effects of temporary water stress after anthesis on grain yield and yield components in different tiller categories of two spring wheat varieties. Journal of Agronomy and Crop Science. 1994;173(1):32–40.

[46] Klepper B, Rickman R, Peterson C. Quantitative characterization of vegetative development in small cereal grains. Agronomy Journal. 1982;74(5):789–92.

[47] Innes P, Blackwell R. The effect of drought on the water use and yield of two spring wheat genotypes. The Journal of Agricultural Science. 1981;96(03):603–10.

[48] Asseng S, Ritchie JT, Smucker AJM, Robertson MJ. Root growth and water uptake during water deficit and recovering in wheat. Plant and Soil. 1998;201:265–73.

[49] Wise K, Johnson B, Mansfield C, Krupke C. Managing wheat by growth stage (ID-422): Purdue University; 2011. Available: https://www.extension.purdue.edu/extmedia/ID/ID-422.pdf. Accessed 16 February 2015.

[50] Monneveux P, Jing R, Misra SC. Phenotyping for drought adaptation in wheat using physiological traits. Frontiers in Physiology. 2012;3 10.3389/fphys.2012.00429 PMC3499878 23181021

[51] Monneveux P, Jing R, Misra SC. Phenotyping wheat for adaptation to drought Drought phenotyping in crops: from theory to practice: CGIAR Generation Challenge Program; 2011.

[52] Wang L, D'Odorico P, Evans J, Eldridge D, McCabe M, Caylor K, et al Dryland ecohydrology and climate change: critical issues and technical advances. Hydrology and Earth System Sciences. 2012;16:2585–603.

[53] van Heerwaarden J, Doebley J, Briggs WH, Glaubitz JC, Goodman MM, Gonzalez JdJS, et al Genetic signals of origin, spread, and introgression in a large sample of maize landraces. Proceedings of the National Academy of Sciences. 2011;108(3):1088–92. 10.1073/pnas.1013011108 PMC3024656 21189301

[54] Haudry A, Cenci A, Ravel C, Bataillon T, Brunel D, Poncet C, et al Grinding up wheat: A massive loss of nucleotide diversity since domestication. Molecular Biology and Evolution. 2007;24(1506–1517). 10.1093/molbev/msm077 17443011

[55] Awika JM. Major Cereal Grains Production and Use around the World In: Awika JM, Piironen V, Bean S, editors. Advances in Cereal Science: Implications to Food Processing and Health Promotion. 1089: American Chemical Society; 2011 p. 1–13.

[56] Shein EV, Pachepsky YA. Influence of root density on the critical soil water potential. Plant and Soil. 1995;171:351–7.

[57] Hamblin AP, Tennant D. Root length density and water uptake in cereals and grain legumes: how well are they correlated. Australian Journal of Agricultural Research. 1987;38:513–27.

[58] Vadez V, Rao S, Kholova J, Krishnamurthi L, Kashiwagi J, Ratnakumar P, et al Root research for drought tolerance in legumes: *Quo vadis*? Journal of Food Legumes. 2008;21(2):77–85.

[59] Hamblin A, Hamblin J. Root characteristics of some temperate legume species and varieties on deep, free-draining entisols. Crop and Pasture Science. 1985;36(1):63–72.

[60] Yue B, Xue W, Xiong L, Yu X, Luo L, Cui K, et al Genetic basis of drought resistance at reproductive stage in rice: separation of drought tolerance from drought avoidance. Genetics. 2006;172(2):1213–28. 16272419 10.1534/genetics.105.045062 PMC1456219

