# Supplementary material for: Global Synthesis of Drought Effects on Maize and Wheat Production
Source: PLoS One. 2016 May 25;11(5):e0156362. doi: 10.1371/journal.pone.0156362 (PMC4880198; doi:10.1371/journal.pone.0156362)
Supplement: S1 Fig — (PDF) [file pone.0156362.s001.pdf]

PRISMA 2009 Flow Diagram

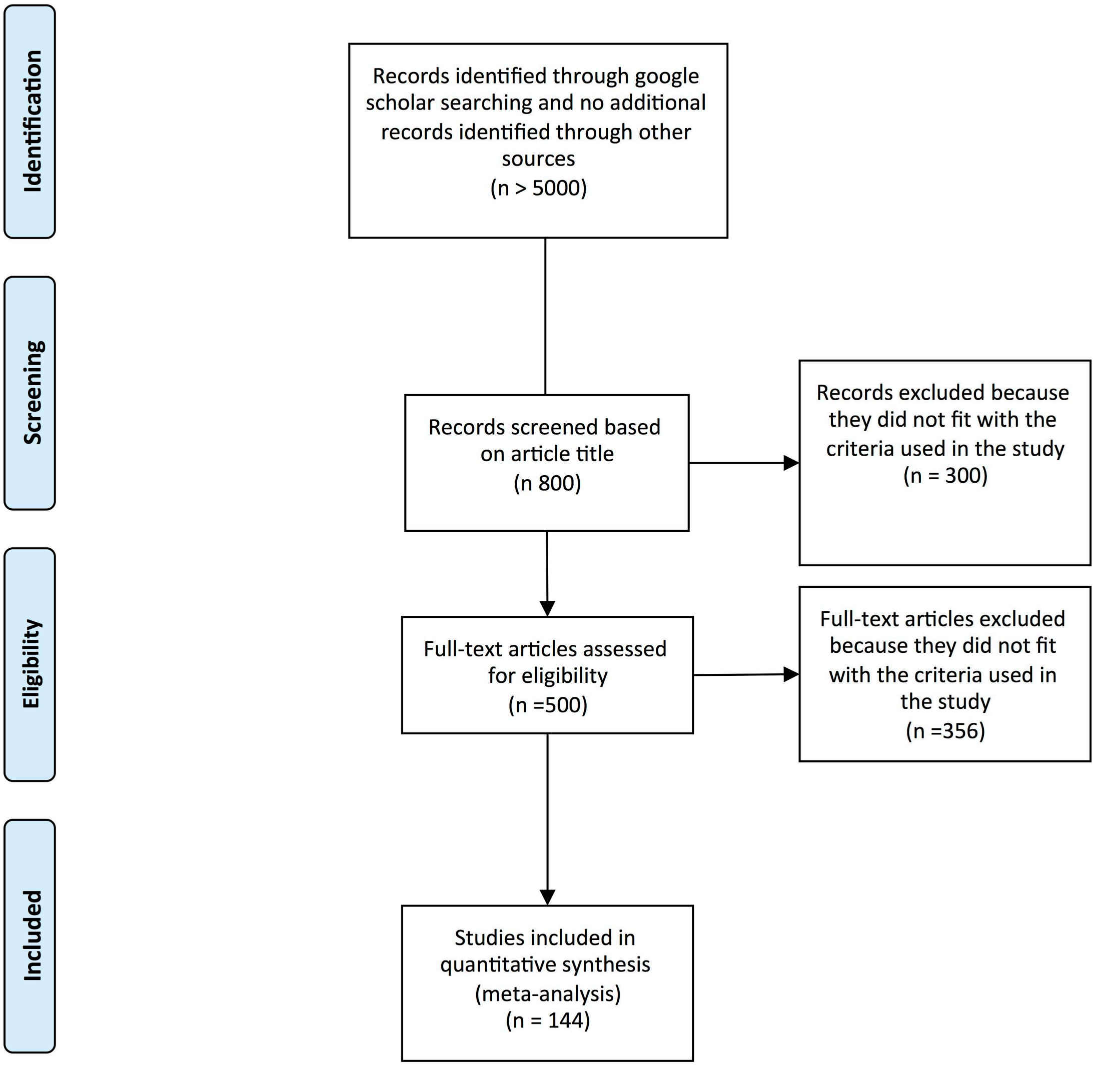

From: Moher D, Liberati A, Tetzlaff J, Altman DG, The PRISMA Group (2009). Preferred Reporting Items for Systematic Reviews and Meta Analysis. The PRISMA Statement 6(6): e1000097. doi: 10.1371/journal.pmed1000097

For more information, visit www.prisma-statement.org
