# Supplementary material for: Global Synthesis of Drought Effects on Maize and Wheat Production
Source: PLoS One. 2016 May 25;11(5):e0156362. doi: 10.1371/journal.pone.0156362 (PMC4880198; doi:10.1371/journal.pone.0156362)
Supplement: S2 Information — (PDF) [file pone.0156362.s007.pdf]

S2 Information

List of references used in this study

1 Abrecht, D. & Carberry, P. The influence of water deficit prior to tassel initiation on maize growth, development and yield. *Field Crop Res.* **31**, 55-69 (1993).

2 Aggarwal, P., Singh, A., Chaturvedi, G. & Sinha, S. Performance of wheat and triticale cultivars in a variable soil—water environment II. Evapotranspiration, water use efficiency, harvest index and grain yield. *Field Crop Res.* **13**, 301-315 (1986).

3 Aggarwal, P. & Sinha, S. Response of droughted wheat to mid-season water application: recovery in leaf area and its effect on grain yield. *Funct. Plant Biol.* **14**, 227-237 (1987).

4 Aguilar, M., Borjas, F. & Espinosa, M. Agronomic response of maize to limited levels of water under furrow irrigation in southern Spain. *Span. J. Agric. Res.* **5**, 587-592 (2007).

5 Ahmad, R., Qadir, S., Ahmad, N. & Shah, K. H. Yield potential and stability of nine wheat varieties under water stress conditions. *Int. J. Agric. Biol* **5**, 7-9 (2003).

6 Akçura, M., Partigoç, F. & Kaya, Y. Evaluating of drought stress tolerance based on selection indices in Turkish bread wheat landraces. *J. Anim. Plant Sci* **21**, 700-709 (2011).

7 Akram, M. Growth and yield components of wheat under water stress of different growth stages. *Bangladesh J. Agric. Res.* **36**, 455-468 (2011).

8 Ali, M. H., Hoque, M., Hassan, A. & Khair, A. Effects of deficit irrigation on yield, water productivity, and economic returns of wheat. *Agric. Water Manage.* **92**, 151-161 (2007).

9 Andarzian, B. *et al.* Validation and testing of the AquaCrop model under full and deficit irrigated wheat production in Iran. *Agric. Water Manage.* **100**, 1-8 (2011).

10 Araghi, S. G. & Assad, M. Evaluation of four screening techniques for drought resistance and their relationship to yield reduction ratio in wheat. *Euphytica* **103**, 293-299 (1998).

11 Bayoumi, T., Eid, M. H. & Metwali, E. Application of physiological and biochemical indices as a screening technique for drought tolerance in wheat genotypes. *Afr. J. Biotechnol.* **7** (2008).

12 Bolaños, J. & Edmeades, G. Eight cycles of selection for drought tolerance in lowland tropical maize. I. Responses in grain yield, biomass, and radiation utilization. *Field Crop Res.* **31**, 233-252 (1993).

13 Butler, J. D., Byrne, P. F., Mohammadi, V., Chapman, P. L. & Haley, S. D. Agronomic Performance of Alleles in a Spring Wheat Population across a Range of Moisture Levels. *Crop Sci.* **45**, 939-947 (2005).

14 Çakir, R. Effect of water stress at different development stages on vegetative and reproductive growth of corn. *Field Crop Res.* **89**, 1-16 (2004).

15 Carberry, P., Muchow, R. & McCown, R. Testing the CERES-Maize simulation model in a semi-arid tropical environment. *Field Crop Res.* **20**, 297-315 (1989).

16 Chaturvedi, G., Aggarwal, P., Singh, A., Joshi, M. & Sinha, S. Effect of irrigation on tillering in wheat, triticale and barley in a water-limited environment. *Irrig. Sci.* **2**, 225-235 (1981).

17 Choudhury, P. & Kumar, V. The sensitivity of growth and yield of dwarf wheat to water stress at three growth stages. *Irrig. Sci.* **1**, 223-231 (1980).

18 Da Ge, T. *et al.* Differential responses of yield and selected nutritional compositions to drought stress in summer maize grains. *J. Plant Nutrition* **33**, 1811-1818 (2010).

19 Dadbakhsh, A., Yazdansepas, A. & Ahmadizadeh, M. Study drought stress on yield of wheat (*Triticum aestivum* L.) genotypes by drought tolerance indices. *Adv. Environ. Biol.* **5**, 1804-1810 (2011).

20 de Souza, T. C. *et al.* Morphophysiology, morphoanatomy, and grain yield under field conditions for two maize hybrids with contrasting response to drought stress. *Acta Physiol. Plant.* **35**, 3201-3211 (2013).

21 Du, T., Kang, S., Sun, J., Zhang, X. & Zhang, J. An improved water use efficiency of cereals under temporal and spatial deficit irrigation in north China. *Agric. Water Manage.* **97**, 66-74 (2010).

22 Duggan, B., Domitruk, D. & Fowler, D. Yield component variation in winter wheat grown under drought stress. *Can. J. Plant Sci.* **80**, 739-745 (2000).

23 Edmeades, G., Chapman, S. & Lafitte, H. Selection improves drought tolerance in tropical maize populations: I. Gains in biomass, grain yield, and harvest index. *Crop Sci.* **39**, 1306-1315 (1999).

24 Ehdaie, B. Variation in water-use efficiency and its components in wheat: II. Pot and field experiments. *Crop Sci.* **35**, 1617-1626 (1995).

25 El-Tantawy, M. M., Ouda, S. A. & Khalil, F. A. Irrigation scheduling for maize grown under Middle Egypt conditions. *Res. J. Agr. Biol. Sci.* **3**, 456-462 (2007).

26 English, M. & Nakamura, B. Effects of deficit irrigation and irrigation frequency on wheat yields. *J. Irrig. Drain. Eng.* **115**, 172-184 (1989).

27 Eskandari, H. & KAZEMI, K. K. Response of different bread wheat (*Triticum aestivum* L.) genotypes to post-anthesis water deficit. *Not. Sci. Biol.* **2**, 49-52 (2010).

28 Fapohunda, H., Aina, P. & Hossain, M. Water use—Yield relations for cowpea and maize. *Agric. Water Manage.* **9**, 219-224 (1984).

29 Fardad, H. & Pessarakli, M. Biomass production and water use efficiency of barley and wheat plants with different irrigation intervals at various water levels. *J. Plant Nutr.* **18**, 2643-2654 (1995).

30 Farré, I. & Faci, J.-M. Deficit irrigation in maize for reducing agricultural water use in a Mediterranean environment. *Agric. Water Manage.* **96**, 383-394 (2009).

31 Farré, I. & Faci, J. M. Comparative response of maize (*Zea mays* L.) and sorghum (*Sorghum bicolor* L. Moench) to deficit irrigation in a Mediterranean environment. *Agric. Water Manage.* **83**, 135-143 (2006).

32 Farré, I., Van Oijen, M., Leffelaar, P. & Faci, J. Analysis of maize growth for different irrigation strategies in northeastern Spain. *Eur. J. Agron.* **12**, 225-238 (2000).

33 Farshadfar, E. & Elyasi, P. Screening quantitative indicators of drought tolerance in bread wheat (*Triticum aestivum* L.) landraces. *Eur. J. Exp. Biol.* **2**, 577-584 (2012).

34 Fischer, K., Edmeades, G. & Johnson, E. Selection for the improvement of maize yield under moisture-deficits. *Field Crops Research* **22**, 227-243 (1989).

35 Foulkes, M., Scott, R. & Sylvester-Bradley, R. The ability of wheat cultivars to withstand drought in UK conditions: formation of grain yield. *J. Agric. Sci.* **138**, 153-169 (2002).

36 Gales, K. & Wilson, N. Effects of water shortage on the yield of winter wheat. *Ann. Appl. Biol.* **99**, 323-334 (1981).

37 Gholamin, R. & Khayatnezhad, M. The effect of end season drought stress on the chlorophyll content, chlorophyll fluorescence parameters and yield in maize cultivars. *Sci Res. Essay* **25**, 5351-5357 (2011).

38 Ghooshchi, F., Seilsepour, M. & Jafari, P. Effects of water stress on yield and some agronomic traits of maize [Sc301]. *World J. Agric. Sci.* **4**, 684-687 (2008).

39 Golbashy, M., Ebrahimi, M., Khorasani, S. K. & Choukan, R. Evaluation of drought tolerance of some corn (*Zea mays* L.) hybrids in Iran. *Afr. J. Agric. Res.* **5**, 2714-2719 (2010).

40 Gordon, W., Raney, R. & Stone, L. Irrigation management practices for corn production in north central Kansas. *J. Soil Water Conserv.* **50**, 395-399 (1995).

41 Grzesiak, M. T., Marcińska, I., Janowiak, F., Rzepka, A. & Hura, T. The relationship between seedling growth and grain yield under drought conditions in maize and triticale genotypes. *Acta Physiol. Plant.* **34**, 1757-1764 (2012).

42 Guttieri, M. J., Stark, J. C., O'Brien, K. & Souza, E. Relative sensitivity of spring wheat grain yield and quality parameters to moisture deficit. *Crop Sci.* **41**, 327-335 (2001).

43 Han, H. *et al.* Radiation use efficiency and yield of winter wheat under deficit irrigation in North China. *Plant Soil Environ.* **54**, 313-319 (2008).

44 Harder, H., Carlson, R. & Shaw, R. Yield, yield components, and nutrient content of corn grain as influenced by post-silking moisture stress. *Agron. J.* **74**, 275-278 (1982).

45 Heng, L. K., Hsiao, T., Evett, S., Howell, T. & Steduto, P. Validating the FAO AquaCrop model for irrigated and water deficient field maize. *Agron. J.* **101**, 488-498 (2009).

46 Howell, T., Yazar, A., Schneider, A., Dusek, D. & Copeland, K. Yield and water use efficiency of corn in response to LEPA irrigation. *Transactions of the ASAE* **38**, 1737-1747 (1995).

47 Hu, C., Delgado, J., Zhang, X. & Ma, L. Assessment of groundwater use by wheat (*Triticum aestivum* L.) in the Luancheng Xian region and potential implications for water

conservation in the northwestern North China Plain. *J. Soil Water Conserv.* **60**, 80-88 (2005).

48 Huang, M., Gallichand, J. & Zhong, L. Water–yield relationships and optimal water management for winter wheat in the Loess Plateau of China. *Irrig. Sci.* **23**, 47-54 (2004).

49 Hussain, A. *et al.* Influence of water stress on growth, yield and radiation use efficiency of various wheat cultivars. *Intl. J. Agric. Biol.* **6**, 1074-1079 (2004).

50 Igbadun, H. E., Mahoo, H. F., Tarimo, A. K. & Salim, B. A. Crop water productivity of an irrigated maize crop in Mkoji sub-catchment of the Great Ruaha River Basin, Tanzania. *Agric. Water Manage.* **85**, 141-150 (2006).

51 Igbadun, H. E., Salim, B. A., Tarimo, A. K. & Mahoo, H. F. Effects of deficit irrigation scheduling on yields and soil water balance of irrigated maize. *Irrig. Sci.* **27**, 11-23 (2008).

52 Ilbeyi, A., Ustun, H., Oweis, T., Pala, M. & Benli, B. Wheat water productivity and yield in a cool highland environment: Effect of early sowing with supplemental irrigation. *Agric. Water Manage.* **82**, 399-410 (2006).

53 Imtiyaz, M., Kristensen, K. & Mogensen, V. O. Influence of irrigation on water extraction, evapotranspiration, yield and water use efficiency of spring wheat and barley. *Acta Agr. Scand.* **32**, 263-271 (1982).

54 Innes, P. & Blackwell, R. The effect of drought on the water use and yield of two spring wheat genotypes. *J. Agr. Sci.* **96**, 603-610 (1981).

55 Inthapan, P. & Fukai, S. Growth and yield of rice cultivars under sprinkler irrigation in south-eastern Queensland. 2. Comparison with maize and grain sorghum under wet and dry conditions. *Anim. Prod. Sci.* **28**, 243-248 (1988).

56 Irmak, S., Haman, D. Z. & Bastug, R. Determination of crop water stress index for irrigation timing and yield estimation of corn. *Agron. J.* **92**, 1221-1227 (2000).

57 Jama, A. O. & Ottman, M. J. Timing of the first irrigation in corn and water stress conditioning. *Agron. J.* **85**, 1159-1164 (1993).

58 Jamieson, P., Martin, R. & Francis, G. Drought influences on grain yield of barley, wheat, and maize. *New Zeal. J. Crop Hort. Sci.* **23**, 55-66 (1995).

59 Johari-Pireivatlou, M. Effect of soil water stress on yield and proline content of four wheat lines. *Afr. J. Biotechnol.* **9** (2010).

60 Johnson, R. & Kanemasu, E. The influence of water availability on winter wheat yields. *Canadian J. Plant Sci.* **62**, 831-838 (1982).

61 Kahlown, M. A., Raoof, A., Zubair, M. & Kemper, W. D. Water use efficiency and economic feasibility of growing rice and wheat with sprinkler irrigation in the Indus Basin of Pakistan. *Agric. Water Manage.* **87**, 292-298 (2007).

62 Kamara, A., Menkir, A., Badu-Apraku, B. & Ibikunle, O. The influence of drought stress on growth, yield and yield components of selected maize genotypes. *J. Agr. Sci.* **141**, 43-50 (2003).

63 Kang, S., Shi, W. & Zhang, J. An improved water-use efficiency for maize grown under regulated deficit irrigation. *Field Crop Res.* **67**, 207-214 (2000).

64 Kang, S. *et al.* Effects of limited irrigation on yield and water use efficiency of winter wheat in the Loess Plateau of China. *Agric. Water Manage.* **55**, 203-216 (2002).

65 Kara, T. & Biber, C. Irrigation frequencies and corn (*Zea mays* L.) yield relation in Northern Turkey. *Pak. J. Biol. Sci.* **11**, 123-126 (2008).

66 Karam, F., Breidy, J., Stephan, C. & Rouphael, J. Evapotranspiration, yield and water use efficiency of drip irrigated corn in the Bekaa Valley of Lebanon. *Agric. Water Manage.* **63**, 125-137 (2003).

67 Katerji, N., Van Hoorn, J., Hamdy, A. & Mastrorilli, M. Comparison of corn yield response to plant water stress caused by salinity and by drought. *Agric. Water Manage.* **65**, 95-101 (2004).

68 Khamssi, N. N. Assessment of quantitative drought resistance indices under irrigated and rain-fed conditions in bread wheat genotypes. *Adv. Environ. Biol.* **5**, 2591-2595 (2011).

69 Kirda, C. *et al.* Grain yield response and N-fertiliser recovery of maize under deficit

70 Lamm, F., Manges, H., Stone, L., Khan, A. & Rogers, D. Water requirement of subsurface drip-irrigated corn in northwest Kansas. *T. ASAE* **38**, 441-448 (1995).

71 Li, J., Inanaga, S., Li, Z. & Eneji, A. E. Optimizing irrigation scheduling for winter wheat in the North China Plain. *Agric. Water Manage.* **76**, 8-23 (2005).

72 Li, Q., Liu, M., Zhang, J., Dong, B. & Bai, Q. Biomass accumulation and radiation use efficiency of winter wheat under deficit irrigation regimes. *Plant Soil Environ.* **55**, 85-91 (2009).

73 Liao, L., Zhang, L. & Bengtsson, L. Soil moisture variation and water consumption of spring wheat and their effects on crop yield under drip irrigation. *Irrig. Drain. Syst.* **22**, 253-270 (2008).

74 Liu, H., Yu, L., Luo, Y., Wang, X. & Huang, G. Responses of winter wheat (*Triticum aestivum* L.) evapotranspiration and yield to sprinkler irrigation regimes. *Agric. Water Manage.* **98**, 483-492 (2011).

75 Liu, Y., Li, S., Chen, F., Yang, S. & Chen, X. Soil water dynamics and water use efficiency in spring maize (*Zea mays* L.) fields subjected to different water management practices on the Loess Plateau, China. *Agric. Water Manage.* **97**, 769-775 (2010).

76 Lyon, D., Boa, F. & Arkebauer, T. Water-yield relations of several spring-planted dryland crops following winter wheat. *J. Prod. Agric.* **8**, 281-286 (1995).

77 Maralian, H., Ebadi, A. & Haji-Eghrari, B. Influence of water deficit stress on wheat grain yield and proline accumulation rate. *Afr. J. Agr. Res.* **5**, 286-289 (2010).

78 Mason, S. C., Kathol, D., Eskridge, K. M. & Galusha, T. D. Yield increase has been more rapid for maize than for grain sorghum. *Crop Sci.* **48**, 1560-1568 (2008).

79 Mengü, G. P. & Özgürel, M. An Evaluation of Water-Yield Relations in Maize (*Zea mays* L.) in Turkey. *Pak. J. Biol. Sci.* **11**, 517-524 (2008).

80 Menkir, A., Badu-Apraku, B., Ajala, S. & Ndiaye, A. Response of early maturing maize landraces and improved varieties to moisture deficit and sufficient water supply. *Plant Genet. Resour.* **7**, 205-215 (2009).

81 Miller, D. & Hang, A. N. Deficit, high-frequency sprinkler irrigation of wheat. *Soil Sci. Soc. Am. J.* **46**, 386-389 (1982).

82 Mishra, H., Rathore, T. & Savita, U. Water-use efficiency of irrigated winter maize under cool weather conditions of India. *Irrig. Sci.* **21**, 27-33 (2001).

83 Mishra, H., Rathore, T. & Tomar, V. Root growth, water potential and yield of irrigated wheat. *Irrig. Sci.* **18**, 117-123 (1999).

84 Mogensen, V., Jensen, H. & Rab, M. A. Grain yield, yield components, drought sensitivity and water use efficiency of spring wheat subjected to water stress at various growth stages. *Irrigation Science* **6**, 131-140 (1985).

85 Mogensen, V. & Talukder, M. Grain yield of spring wheat in relation to water stress II. Growth Rate of Grains During Drought. *Cereal Res. Comm.* **15**, 247-253 (1987).

86 Moinuddin, Fischer, R., Sayre, K. & Reynolds, M. Osmotic adjustment in wheat in relation to grain yield under water deficit environments. *Agron. J.* **97**, 1062-1071 (2005).

87 Monneveux, P., Rekika, D., Acevedo, E. & Merah, O. Effect of drought on leaf gas exchange, carbon isotope discrimination, transpiration efficiency and productivity in field grown durum wheat genotypes. *Plant Sci.* **170**, 867-872 (2006).

88 Monneveux, P. *et al.* Relationship between grain yield and carbon isotope discrimination in bread wheat under four water regimes. *Eur. J. Agron.* **22**, 231-242 (2005).

89 Morgan, J. & Condon, A. Water use, grain yield, and osmoregulation in wheat. *Funct. Plant Biol.* **13**, 523-532 (1986).

90 Muchow, R. Comparative productivity of maize, sorghum and pearl millet in a semi-arid tropical environment II. Effect of water deficits. *Field Crop Res.* **20**, 207-219 (1989).

91 Muchow, R. Comparative productivity of maize, sorghum and pearl millet in a semi-arid tropical environment I. Yield potential. *Field Crop Res.* **20**, 191-205 (1989).

92 Mugabe, F. & Nyakatawa, E. Effect of deficit irrigation on wheat and opportunities of growing wheat on residual soil moisture in southeast Zimbabwe. *Agric. Water Manage.* **46**, 111-119 (2000).

93 Musick, J. & Dusek, D. Irrigated corn yield response to water. *T. ASAE* **23**, 92-98, 103 (1980).

94 Najafian, G., Jafarnejad, A., Ghandi, A. & Nikooseresht, R. Adaptive traits related to terminal drought tolerance in hexaploid wheat (*Triticum aestivum* L.) genotypes under field conditions. *Crop Breeding J.* **1**, 55-71 (2011).

95 Naserian, B., Asadi, A. A., Rahimi, M. & Ardakani, M. R. Evaluation of wheat cultivars and mutants for morphological and yield traits and comparing of yield components under irrigated and rain fed conditions. *Asian J. Plant Sci.* **6**, 214-224 (2007).

96 NeSmith, D. & Ritchie, J. Short-and long-term responses of corn to a pre-anthesis soil water deficit. *Agronomy Journal* **84**, 107-113 (1992).

97 Nouri-Ganbalani, A., Nouri-Ganbalani, G. & Hassanpanah, D. Effects of drought stress condition on the yield and yield components of advanced wheat genotypes in Ardabil, Iran. *J. Food, Agric. Environ.* **7**, 228-234 (2009).

98 Öktem, A. Effect of Different Irrigation Intervals to Drip Irrigated Dent Corn (*Zea mays* L. indentata) Water-yield Relationship. *Pak. J. Biol. Sci.* **9**, 1476-1481 (2006).

99 Otegui, M. E., Andrade, F. H. & Suero, E. E. Growth, water use, and kernel abortion of maize subjected to drought at silking. *Field Crop Res.* **40**, 87-94 (1995).

100 Ozturk, A. & Aydin, F. Effect of water stress at various growth stages on some quality characteristics of winter wheat. *J. Agron. Crop Sci.* **190**, 93-99 (2004).

101 Pan, X.-Y., Wang, G.-X., Yang, H.-M. & Wei, X.-P. Effect of water deficits on within-plot variability in growth and grain yield of spring wheat in northwest China. *Field Crop Res.* **80**, 195-205 (2003).

102 Panda, R., Behera, S. & Kashyap, P. S. Effective management of irrigation water for wheat under stressed conditions. *Agric. Water Manage.* **63**, 37-56 (2003).

103 Payero, J. O., Klocke, N. L., Schneekloth, J. P. & Davison, D. R. Comparison of irrigation strategies for surface-irrigated corn in West Central Nebraska. *Irrig. Sci.* **24**, 257-265 (2006).

104 Payero, J. O., Melvin, S. R., Irmak, S. & Tarkalson, D. Yield response of corn to deficit irrigation in a semiarid climate. *Agric. Water Manage.* **84**, 101-112 (2006).

105 Payero, J. O., Tarkalson, D. D., Irmak, S., Davison, D. & Petersen, J. L. Effect of irrigation amounts applied with subsurface drip irrigation on corn evapotranspiration, yield, water use efficiency, and dry matter production in a semiarid climate. *Agric. Water Manage.* **95**, 895-908 (2008).

106 Pireivatlou, A. S., Masjedlou, B. D. & Aliyev, R. T. Evaluation of yield potential and stress adaptive trait in wheat genotypes under post anthesis drought stress conditions. *Afr. J. Agric. Res.* **5**, 2829-2836 (2010).

107 Powell, N. L. & Wright, F. S. Grain yield of subsurface microirrigated corn as affected by irrigation line spacing. *Agron. J.* **85**, 1164-1169 (1993).

108 Pradhan, G. P., Xue, Q., Liu, S., Rudd, J. C. & Jessup, K. E. Effective use of soil water contributed to high yield in wheat in the US Southern High Plains. *J. Arid Land* **24**, 153-156 (2014).

109 Qadir, G., Saeed, M. & Cheema, M. A. Effect of water stress on growth and yield performance of four wheat cultivars. *Pak. J. Biol. Sci* **2**, 236-239 (1999).

110 Rahman, S., Talukdar, S., Kaul, A. & Biswas, M. Yield response of a semi-dwarf wheat variety to irrigation on a calcareous brown flood plain soil of Bangladesh. *Agric. Water Manage.* **3**, 217-225 (1981).

111 Retta, A. & Hanks, R. Corn and alfalfa production as influenced by limited irrigation. *Irrig. Sci.* **1**, 135-147 (1980).

112 Saint Pierre, C., Crossa, J. L., Bonnett, D., Yamaguchi-Shinozaki, K. & Reynolds, M. P. Phenotyping transgenic wheat for drought resistance. *J. Exp. Bot.*, err385 (2012).

113 Salemi, H. *et al.* Application of AquaCrop model in deficit irrigation management of winter wheat in arid region. *Afr. J. Agr. Res.* **6**, 2204-2215 (2011).

114 Sharma, D., Kumar, A. & Singh, K. Effect of irrigation scheduling on growth, yield and evapotranspiration of wheat in sodic soils. *Agric. Water Manage.* **18**, 267-276 (1990).

115 Simsek, M., Can, A., Denek, N. & Tonkaz, T. The effects of different irrigation regimes on yield and silage quality of corn under semi-arid conditions. *Afr. J. Biotechnol.* **10**, 5869-5877 (2013).

116 Singh, B. & Singh, D. Agronomic and physiological responses of sorghum, maize and pearl millet to irrigation. *Field Crop Res.* **42**, 57-67 (1995).

117 Singh, P., Wolkewitz, H. & Kumar, R. Comparative performance of different crop production functions for wheat (*Triticum aestivum* L.). *Irrig. Sci.* **8**, 273-290 (1987).

118 Singh, S., Gupta, A. & Kaur, N. Differential Responses of Antioxidative Defence System to Long-Term Field Drought in Wheat (*Triticum aestivum* L.) Genotypes Differing in Drought Tolerance. *J. Agron. Crop Sci.* **198**, 185-195 (2012).

119 Singh, T. & Malik, D. Effect of water stress at three growth stages on the yield and water-use efficiency of dwarf wheat. *Irrig. Sci.* **4**, 239-245 (1983).

120 Sobrado, M. Drought responses of tropical corn. 1. Leaf area and yield components in the field. *Maydica* **35**, 221-226 (1990).

121 Soler, C., Hoogenboom, G., Sentelhas, P. & Duarte, A. P. Impact of Water Stress on Maize Grown Off-Season in a Subtropical Environment. *J. Agron. Crop Sci.* **193**, 247-261 (2007).

122 Steele, D., Stegman, E. & Gregor, B. Field comparison of irrigation scheduling methods for corn. *T. ASAE* **37**, 1197-1197 (1994).

123 Stegman, E. Corn grain yield as influenced by timing of evapotranspiration deficits. *Irrig. Sci.* **3**, 75-87 (1982).

124 Steiner, J., Smith, R., Meyer, W. & Adeney, J. Water use, foliage temperature and yield of irrigated wheat in south-eastern Australia. *Crop Pasture Sci.* **36**, 1-11 (1985).

125 Sun, H.-Y., Liu, C.-M., Zhang, X.-Y., Shen, Y.-J. & Zhang, Y.-Q. Effects of irrigation on water balance, yield and WUE of winter wheat in the North China Plain. *Agric. Water Manage.* **85**, 211-218 (2006).

126 Talukder, M., Mogensen, V. & Jensen, H. Grain yield of spring wheat in relation to water stress: I. Effect of Early Drought on Devellopment of Late Tillers. *Cereal Res. Comm.* **15**, 101-107 (1987).

127 Thompson, J. & Chase, D. Effect of limited irrigation on growth and yield of a semi-dwarf wheat in southern New South Wales. *Anim. Prod. Sci.* **32**, 725-730 (1992).

128 Tolk, J. A., Howell, T. A. & Evett, S. R. Evapotranspiration and yield of corn grown on three high plains soils. *Agron. J.* **90**, 447-454 (1998).

129 van Donk, S. J., Petersen, J. L. & Davison, D. R. Effect of amount and timing of subsurface drip irrigation on corn yield. *Irrig. Sci.* **31**, 599-609 (2013).

130 Vories, E., Tacker, P., Lancaster, S. & Glover, R. Subsurface drip irrigation of corn in the United States Mid-South. *Agric. Water Manage.* **96**, 912-916 (2009).

131 Weerathaworn, P., Thiraporn, R., Soldati, A. & Stamp, P. Yield and agronomic characters of tropical maize (*Zea mays* L.) cultivars under different irrigation regimes. *J. Agron. Crop Sci.* **168**, 326-336 (1992).

132 Wenda, W. & Hanks, R. Corn yield and evapotranspiration under simulated drought conditions. *Irrig. Sci.* **2**, 193-204 (1981).

133 Yazar, A., Gökçel, F. & Sezen, M. Corn yield response to partial rootzone drying and deficit irrigation strategies applied with drip system. *Plant Soil Environ.* **55**, 494-503 (2009).

134 Yazar, A., Howell, T., Dusek, D. & Copeland, K. Evaluation of crop water stress index for LEPA irrigated corn. *Irrig. Sci.* **18**, 171-180 (1999).

135 Yi, L., Shenjiao, Y., Shiqing, L., Xinping, C. & Fang, C. Growth and development of maize (*Zea mays* L.) in response to different field water management practices: Resource capture and use efficiency. *Agr. Forest Meteorol.* **150**, 606-613 (2010).

136 Zarea-Fizabady, A. & Ghodsi, M. Evaluation of yield and yield components of facultative and winter bread wheat genotypes (*Triticum aestivum* L.) under different irrigation regimes in Khorasam Province in Iran. *J. Agron* **3**, 184-187 (2004).

137 Zhang, B., Li, F.M., Huang, G., Cheng, Z.Y. & Zhang, Y. Yield performance of spring wheat improved by regulated deficit irrigation in an arid area. *Agric. Water Manage.* **79**, 28-42 (2006).

138 Zhang, B.C. *et al.* Effects of regulated deficit irrigation on grain yield and water use efficiency of spring wheat in an arid environment. *Can. J. Plant Sci.* **85**, 829-837 (2005).

139 Zhang, H. & Oweis, T. Water–yield relations and optimal irrigation scheduling of wheat in the Mediterranean region. *Agric. Water Manage.* **38**, 195-211 (1999).

140 Zhang, H., Wang, X., You, M. & Liu, C. Water-yield relations and water-use efficiency of winter wheat in the North China Plain. *Irrig. Sci.* **19**, 37-45 (1999).

141 Zhang, J. *et al.* An improved water-use efficiency for winter wheat grown under reduced irrigation. *Field Crop Res.* **59**, 91-98 (1998).

142 Zhang, X., Chen, S., Liu, M., Pei, D. & Sun, H. Improved water use efficiency associated with cultivars and agronomic management in the North China Plain. *Agron. J.* **97**, 783-790 (2005).

143 Zhang, X., Chen, S., Sun, H., Pei, D. & Wang, Y. Dry matter, harvest index, grain yield and water use efficiency as affected by water supply in winter wheat. *Irrig. Sci.* **27**, 1-10 (2008).

144 Zhang, Y. *et al.* Effect of soil water deficit on evapotranspiration, crop yield, and water use efficiency in the North China Plain. *Agric. Water Manage.* **64**, 107-122 (2004).
