# Supplementary material for: Global Synthesis of Drought Effects on Maize and Wheat Production
Source: PLoS One. 2016 May 25;11(5):e0156362. doi: 10.1371/journal.pone.0156362 (PMC4880198; doi:10.1371/journal.pone.0156362)
Supplement: S1 Table — (PDF) [file pone.0156362.s008.pdf]

type of plant	ratio of mean productivity during drought and during normal condition	country	continent	dryland/non dryland agriculture (based on Koppen or	drought timing	ratio of water during drought and during normal condition	soil texture	author(s)	year of publication
wheat	0.61	new	australia	non-dryland	vegetative	0.62	coarse	jamieson et al	1995
wheat	0.63	new	australia	non-dryland	generative	0.58	coarse	jamieson et al	1995
maize	0.87	new	australia	non-dryland	vegetative	0.77	coarse	jamieson et al	1995
maize	0.79	new	australia	non-dryland	generative	0.59	coarse	jamieson et al	1995
maize	1.09	new	australia	non-dryland	generative	0.63	coarse	jamieson et al	1995
wheat	0.57	egypt	africa	dryland	throughout	0.33	n/a	bayoumi et al	2008
maize	0.38	nigeria	africa	dryland	throughout	n/a	fine	kamara et al	2003
maize	0.86	USA	north america	dryland	throughout	0.61	medium	wenda & hanks	1981
maize	1.00	USA	north america	dryland	throughout	0.60	medium	wenda & hanks	1981
maize	0.78	USA	north america	dryland	throughout	0.61	medium	wenda & hanks	1981
maize	0.77	USA	north america	dryland	throughout	0.58	medium	wenda & hanks	1981
maize	0.70	USA	north america	dryland	throughout	0.38	medium	wenda & hanks	1981
maize	1.01	USA	north america	dryland	generative	0.86	medium	wenda & hanks	1981
maize	1.06	USA	north america	dryland	generative	0.84	medium	wenda & hanks	1981
maize	0.97	USA	north america	dryland	generative	0.85	medium	wenda & hanks	1981
maize	0.96	USA	north america	dryland	generative	0.87	medium	wenda & hanks	1981
maize	1.00	USA	north america	dryland	generative	0.71	medium	wenda & hanks	1981
maize	0.72	USA	north america	dryland	throughout	0.47	coarse	wenda & hanks	1981
maize	0.60	USA	north america	dryland	throughout	0.47	coarse	wenda & hanks	1981
maize	0.51	USA	north america	dryland	throughout	0.48	n/a	wenda & hanks	1981
maize	0.53	USA	north america	dryland	throughout	0.46	coarse	wenda & hanks	1981
maize	0.71	USA	north america	dryland	throughout	0.35	coarse	wenda & hanks	1981
maize	0.91	USA	north america	dryland	throughout	0.75	n/a	wenda & hanks	1981
maize	0.82	USA	north america	dryland	generative	0.75	coarse	wenda & hanks	1981
maize	0.72	USA	north america	dryland	generative	0.76	coarse	wenda & hanks	1981
maize	0.80	USA	north america	dryland	generative	0.73	n/a	wenda & hanks	1981
maize	0.91	USA	north america	dryland	generative	0.67	coarse	wenda & hanks	1981
maize	0.39	USA	north america	dryland	throughout	0.37	medium	wenda & hanks	1981
maize	0.37	USA	north america	dryland	throughout	0.39	medium	wenda & hanks	1981
maize	0.33	USA	north america	dryland	throughout	0.38	medium	wenda & hanks	1981
maize	0.26	USA	north america	dryland	throughout	0.41	medium	wenda & hanks	1981
maize	0.36	USA	north america	dryland	throughout	0.39	medium	wenda & hanks	1981
maize	0.88	USA	north america	dryland	generative	0.69	medium	wenda & hanks	1981
maize	0.79	USA	north america	dryland	generative	0.70	medium	wenda & hanks	1981
maize	0.69	USA	north america	dryland	generative	0.71	medium	wenda & hanks	1981
maize	0.56	USA	north america	dryland	generative	0.72	medium	wenda & hanks	1981
maize	0.61	USA	north america	dryland	generative	0.70	medium	wenda & hanks	1981
wheat	0.79	iran	asia	dryland	throughout	0.48	n/a	araghi & assad	1998
wheat	0.50	iran	asia	dryland	generative	0.67	medium	nouri-ganbalani et al	2009
maize	0.74	lebanon	asia	dryland	throughout	0.60	fine	karam et al	2003
maize	0.81	lebanon	asia	dryland	throughout	0.60	fine	karam et al	2003
wheat	1.38	china	asia	dryland	generative	0.87	medium	zhang et al	2006
wheat	1.36	china	asia	dryland	generative	0.77	medium	zhang et al	2006
wheat	1.36	china	asia	dryland	vegetative	0.85	medium	zhang et al	2006
wheat	1.15	china	asia	dryland	vegetative	0.85	medium	zhang et al	2006
wheat	1.03	china	asia	dryland	generative	0.77	medium	zhang et al	2006
wheat	1.00	china	asia	dryland	vegetative	0.81	medium	zhang et al	2006
maize	0.96	turkey	asia	dryland	vegetative	0.83	medium	cakir	2004
maize	0.72	turkey	asia	dryland	generative	0.71	medium	cakir	2004
maize	0.70	turkey	asia	dryland	generative	0.70	medium	cakir	2004
maize	0.75	turkey	asia	dryland	generative	0.76	medium	cakir	2004
maize	0.26	turkey	asia	dryland	generative	0.17	medium	cakir	2004
maize	0.43	turkey	asia	dryland	throughout	0.29	medium	cakir	2004
maize	0.51	turkey	asia	dryland	throughout	0.30	medium	cakir	2004
maize	0.35	turkey	asia	dryland	throughout	0.24	medium	cakir	2004
maize	0.88	turkey	asia	dryland	vegetative	0.78	medium	cakir	2004
maize	0.51	turkey	asia	dryland	generative	0.68	medium	cakir	2004
maize	0.74	turkey	asia	dryland	generative	0.74	medium	cakir	2004
maize	0.72	turkey	asia	dryland	generative	0.76	medium	cakir	2004
maize	0.07	turkey	asia	dryland	generative	0.19	medium	cakir	2004
maize	0.44	turkey	asia	dryland	throughout	0.32	medium	cakir	2004
maize	0.37	turkey	asia	dryland	throughout	0.26	medium	cakir	2004
maize	0.10	turkey	asia	dryland	throughout	0.24	medium	cakir	2004
maize	0.89	turkey	asia	dryland	vegetative	0.85	medium	cakir	2004
maize	0.85	turkey	asia	dryland	generative	0.61	medium	cakir	2004
maize	0.83	turkey	asia	dryland	generative	0.76	medium	cakir	2004
maize	0.86	turkey	asia	dryland	generative	0.78	medium	cakir	2004
maize	0.56	turkey	asia	dryland	generative	0.15	medium	cakir	2004
maize	0.79	turkey	asia	dryland	throughout	0.39	medium	cakir	2004
maize	0.71	turkey	asia	dryland	throughout	0.24	medium	cakir	2004
maize	0.71	turkey	asia	dryland	throughout	0.22	medium	cakir	2004
wheat	0.86	india	asia	dryland	vegetative	n/a	coarse	singh & malik	1983
wheat	0.76	india	asia	dryland	vegetative	n/a	coarse	singh & malik	1983
wheat	0.65	india	asia	dryland	vegetative	n/a	coarse	singh & malik	1983
wheat	0.90	india	asia	dryland	vegetative	n/a	coarse	singh & malik	1983

wheat	0.82	india	asia	dryland	generative	n/a	coarse	singh & malik	1983
wheat	0.72	india	asia	dryland	generative	n/a	coarse	singh & malik	1983
wheat	0.94	india	asia	dryland	generative	n/a	coarse	singh & malik	1983
wheat	0.84	india	asia	dryland	generative	n/a	coarse	singh & malik	1983
wheat	0.75	india	asia	dryland	generative	n/a	coarse	singh & malik	1983
wheat	0.87	india	asia	dryland	vegetative	n/a	coarse	singh & malik	1983
wheat	0.76	india	asia	dryland	vegetative	n/a	coarse	singh & malik	1983
wheat	0.66	india	asia	dryland	vegetative	n/a	coarse	singh & malik	1983
wheat	0.91	india	asia	dryland	vegetative	n/a	coarse	singh & malik	1983
wheat	0.83	india	asia	dryland	generative	n/a	coarse	singh & malik	1983
wheat	0.74	india	asia	dryland	generative	n/a	coarse	singh & malik	1983
wheat	0.96	india	asia	dryland	generative	n/a	coarse	singh & malik	1983
wheat	0.86	india	asia	dryland	generative	n/a	coarse	singh & malik	1983
wheat	0.79	india	asia	dryland	generative	n/a	coarse	singh & malik	1983
wheat	0.74	iran	asia	dryland	generative	n/a	medium	johari-pirevatlou	2010
wheat	0.70	iran	asia	dryland	generative	n/a	coarse	ahmad et al	2003
maize	1.04	turkey	asia	dryland	throughout	0.88	fine	oktem	2006
maize	0.84	turkey	asia	dryland	throughout	0.82	fine	oktem	2006
maize	0.76	turkey	asia	dryland	throughout	0.73	fine	oktem	2006
maize	1.08	turkey	asia	dryland	throughout	0.91	fine	oktem	2006
maize	0.92	turkey	asia	dryland	throughout	0.78	fine	oktem	2006
maize	0.78	turkey	asia	dryland	throughout	0.70	fine	oktem	2006
maize	1.01	china	asia	dryland	throughout	0.82	medium	zhang et al	2004
maize	0.57	china	asia	dryland	throughout	0.76	medium	zhang et al	2004
wheat	1.09	china	asia	dryland	generative	0.62	medium	zhang et al	2004
wheat	1.05	china	asia	dryland	generative	0.66	medium	zhang et al	2004
wheat	0.92	china	asia	dryland	generative	0.71	medium	zhang et al	2004
wheat	0.98	china	asia	dryland	vegetative	0.62	medium	zhang et al	2004
wheat	1.07	china	asia	dryland	vegetative	0.66	medium	zhang et al	2004
wheat	0.96	china	asia	dryland	vegetative	0.71	medium	zhang et al	2004
wheat	0.99	china	asia	dryland	vegetative	0.62	medium	zhang et al	2004
wheat	1.03	china	asia	dryland	vegetative	0.66	medium	zhang et al	2004
wheat	0.94	china	asia	dryland	vegetative	0.71	medium	zhang et al	2004
maize	0.95	USA	north america	dryland	throughout	0.86	medium	payero et al	2008
maize	1.04	USA	north america	dryland	throughout	0.62	medium	payero et al	2008
maize	0.98	USA	north america	dryland	throughout	0.62	medium	payero et al	2008
maize	0.90	USA	north america	dryland	throughout	0.43	medium	payero et al	2008
maize	0.90	USA	north america	dryland	throughout	0.29	medium	payero et al	2008
maize	0.87	USA	north america	dryland	throughout	0.21	medium	payero et al	2008
maize	0.81	USA	north america	dryland	throughout	0.15	medium	payero et al	2008
maize	0.99	USA	north america	dryland	throughout	0.87	medium	payero et al	2008
maize	1.03	USA	north america	dryland	throughout	0.77	medium	payero et al	2008
maize	1.02	USA	north america	dryland	throughout	0.81	medium	payero et al	2008
maize	0.94	USA	north america	dryland	throughout	0.58	medium	payero et al	2008
maize	0.87	USA	north america	dryland	throughout	0.43	medium	payero et al	2008
maize	0.76	USA	north america	dryland	throughout	0.29	medium	payero et al	2008
maize	0.49	USA	north america	dryland	throughout	0.10	medium	payero et al	2008
maize	0.00	australia	australia	non-dryland	generative	0.58	medium	muchow	1989
maize	0.11	australia	australia	non-dryland	generative	0.67	medium	muchow	1989
maize	0.96	australia	australia	non-dryland	generative	0.85	medium	muchow	1989
maize	0.73	australia	australia	non-dryland	generative	0.42	medium	muchow	1989
maize	0.68	australia	australia	non-dryland	vegetative	0.52	medium	muchow	1989
maize	0.22	australia	australia	non-dryland	generative	0.22	medium	muchow	1989
maize	0.81	spain	europe	dryland	throughout	0.86	coarse	farre & faci	2006
maize	0.44	spain	europe	dryland	throughout	0.68	coarse	farre & faci	2006
maize	0.18	spain	europe	dryland	throughout	0.50	coarse	farre & faci	2006
maize	0.05	spain	europe	dryland	throughout	0.18	coarse	farre & faci	2006
maize	0.01	spain	europe	dryland	throughout	0.36	coarse	farre & faci	2006
maize	0.81	spain	europe	dryland	throughout	0.86	coarse	farre & faci	2006
maize	0.51	spain	europe	dryland	throughout	0.68	coarse	farre & faci	2006
maize	0.33	spain	europe	dryland	throughout	0.50	coarse	farre & faci	2006
maize	0.23	spain	europe	dryland	throughout	0.18	coarse	farre & faci	2006
maize	0.17	spain	europe	dryland	throughout	0.36	coarse	farre & faci	2006
maize	0.84	argentina	south america	non-dryland	generative	n/a	medium	otegui et al	1995
maize	0.89	argentina	south america	non-dryland	generative	n/a	medium	otegui et al	1995
maize	0.83	argentina	south america	non-dryland	generative	n/a	medium	otegui et al	1995
maize	0.96	argentina	south america	non-dryland	generative	n/a	medium	otegui et al	1995
maize	0.84	australia	australia	non-dryland	vegetative	0.90	medium	abretch & carberry	1993
maize	1.00	australia	australia	non-dryland	vegetative	0.77	medium	abretch & carberry	1993
maize	0.93	australia	australia	non-dryland	vegetative	0.59	medium	abretch & carberry	1993
maize	0.01	USA	north america	non-dryland	generative	0.96	coarse	nesmith & ritchie	1992
maize	0.10	USA	north america	non-dryland	generative	0.81	coarse	nesmith & ritchie	1992
maize	0.00	USA	north america	non-dryland	generative	0.60	coarse	nesmith & ritchie	1992
maize	0.77	USA	north america	dryland	throughout	0.84	medium	lyod et al	1995
maize	0.89	USA	north america	dryland	throughout	0.79	medium	lyod et al	1995
maize	0.58	india	asia	dryland	throughout	0.69	coarse	singh & singh	1995
maize	0.52	india	asia	dryland	throughout	0.46	coarse	singh & singh	1995
maize	0.33	india	asia	dryland	throughout	0.27	coarse	singh & singh	1995
wheat	0.77	india	asia	dryland	vegetative	0.96	coarse	choudury & kumar	1980
wheat	0.80	india	asia	dryland	vegetative	0.93	coarse	choudury & kumar	1980

wheat	0.85	india	asia	dryland	generative	0.92	coarse	choudury & kumar	1980
wheat	0.83	india	asia	dryland	generative	0.91	coarse	choudury & kumar	1980
wheat	0.95	india	asia	dryland	generative	0.95	coarse	choudury & kumar	1980
wheat	0.76	india	asia	dryland	generative	0.94	coarse	choudury & kumar	1980
wheat	0.80	india	asia	dryland	vegetative	0.96	coarse	choudury & kumar	1980
wheat	0.67	india	asia	dryland	vegetative	0.93	coarse	choudury & kumar	1980
wheat	0.86	india	asia	dryland	generative	0.92	coarse	choudury & kumar	1980
wheat	0.90	india	asia	dryland	generative	0.91	coarse	choudury & kumar	1980
wheat	0.69	india	asia	dryland	generative	0.95	coarse	choudury & kumar	1980
wheat	0.76	india	asia	dryland	generative	0.94	coarse	choudury & kumar	1980
maize	0.88	turkey	asia	non-dryland	throughout	0.79	fine	irmak et al	2000
maize	0.75	turkey	asia	non-dryland	throughout	0.83	fine	irmak et al	2000
maize	0.12	turkey	asia	non-dryland	throughout	0.15	fine	irmak et al	2000
wheat	0.82	china	asia	non-dryland	throughout	0.50	medium	zhang et al	1998
wheat	0.50	china	asia	non-dryland	throughout	0.33	medium	zhang et al	1998
wheat	0.92	china	asia	non-dryland	throughout	0.49	medium	zhang et al	1998
wheat	0.55	china	asia	non-dryland	throughout	0.32	medium	zhang et al	1998
maize	0.79	USA	north america	non-dryland	vegetative	0.71	coarse	NeSmith & Ritchie	1992c
maize	0.87	USA	north america	non-dryland	vegetative	0.79	coarse	NeSmith & Ritchie	1992c
maize	0.75	italy	europe	dryland	throughout	n/a	medium	katerji et al	2004
maize	0.55	italy	europe	dryland	throughout	n/a	medium	katerji et al	2004
maize	0.83	italy	europe	dryland	throughout	n/a	medium	katerji et al	2004
maize	0.60	italy	europe	dryland	throughout	n/a	medium	katerji et al	2004
wheat	0.80	india	asia	dryland	throughout	0.47	n/a	aggarwal et al	1986
maize	0.71	mexico	south america	dryland	throughout	n/a	n/a	fischer et al	1989
maize	0.25	mexico	south america	dryland	throughout	n/a	n/a	fischer et al	1989
maize	0.52	australia	australia	non-dryland	throughout	0.20	n/a	inthapan & fukai	1988
maize	0.73	USA	north america	non-dryland	generative	0.88	fine	harder et al	1982
maize	0.74	USA	north america	non-dryland	generative	0.90	fine	harder et al	1982
maize	0.67	USA	north america	non-dryland	generative	0.81	fine	harder et al	1982
maize	0.67	USA	north america	non-dryland	generative	0.81	fine	harder et al	1982
maize	0.64	USA	north america	non-dryland	generative	0.78	fine	harder et al	1982
maize	0.59	USA	north america	non-dryland	generative	0.72	fine	harder et al	1982
maize	0.85	USA	north america	non-dryland	generative	0.85	fine	harder et al	1982
maize	0.69	USA	north america	non-dryland	generative	0.70	fine	harder et al	1982
maize	0.69	USA	north america	non-dryland	generative	0.69	fine	harder et al	1982
wheat	0.76	iran	asia	dryland	vegetative	n/a	medium	maralian et al	2010
wheat	0.53	iran	asia	dryland	generative	n/a	medium	maralian et al	2010
maize	0.98	nigeria	africa	non-dryland	throughout	0.88	medium	fapohunda et al	1984
maize	0.84	nigeria	africa	non-dryland	throughout	0.80	medium	fapohunda et al	1984
maize	0.68	nigeria	africa	non-dryland	throughout	0.76	medium	fapohunda et al	1984
maize	0.53	nigeria	africa	non-dryland	throughout	0.72	medium	fapohunda et al	1984
maize	0.45	nigeria	africa	non-dryland	throughout	0.71	medium	fapohunda et al	1984
wheat	0.31	turkey	asia	dryland	throughout	n/a	medium	ackura et al	2011
wheat	0.56	turkey	asia	dryland	throughout	n/a	medium	ackura et al	2011
wheat	0.22	USA	north america	dryland	throughout	0.60	medium	pradhan et al	2014
wheat	0.20	USA	north america	dryland	throughout	0.80	medium	pradhan et al	2014
wheat	0.82	pakistan	asia	dryland	vegetative	n/a	n/a	akram	2011
wheat	0.92	pakistan	asia	dryland	generative	n/a	n/a	akram	2011
wheat	0.78	pakistan	asia	dryland	generative	n/a	n/a	akram	2011
maize	0.66	poland	europe	non-dryland	throughout	0.36	medium	Grzeiak et al	2012
maize	0.97	USA	north america	dryland	throughout	0.88	medium	Lamm et al	1995
maize	0.88	USA	north america	dryland	throughout	0.73	medium	Lamm et al	1995
maize	0.79	USA	north america	dryland	throughout	0.52	medium	Lamm et al	1995
maize	0.70	USA	north america	dryland	throughout	0.49	medium	Lamm et al	1995
maize	0.87	USA	north america	dryland	throughout	0.90	medium	Lamm et al	1995
maize	0.76	USA	north america	dryland	throughout	0.58	medium	Lamm et al	1995
maize	0.57	USA	north america	dryland	throughout	0.51	medium	Lamm et al	1995
maize	0.43	USA	north america	dryland	throughout	0.47	medium	Lamm et al	1995
maize	0.97	USA	north america	dryland	throughout	0.93	medium	Lamm et al	1995
maize	0.74	USA	north america	dryland	throughout	0.57	medium	Lamm et al	1995
maize	0.46	USA	north america	dryland	throughout	0.51	medium	Lamm et al	1995
maize	0.29	USA	north america	dryland	throughout	0.44	medium	Lamm et al	1995
wheat	0.71	iran	asia	dryland	throughout	n/a	medium	fadsafar & elyasi	2012
wheat	0.88	zimbabwe	africa	dryland	throughout	0.82	fine	mugabe & nyakatawa	2000
wheat	0.80	zimbabwe	africa	dryland	throughout	0.63	fine	mugabe & nyakatawa	2000
wheat	0.93	zimbabwe	africa	dryland	throughout	0.82	fine	mugabe & nyakatawa	2000
wheat	0.80	zimbabwe	africa	dryland	throughout	0.63	fine	mugabe & nyakatawa	2000
wheat	0.71	iran	asia	dryland	throughout	0.46	n/a	najafian et al	2011
wheat	0.48	iran	asia	dryland	throughout	0.21	n/a	najafian et al	2011
wheat	0.83	UK	europe	non-dryland	throughout	0.64	coarse	foulkes et al	2002
wheat	0.66	UK	europe	non-dryland	throughout	0.49	coarse	foulkes et al	2002
wheat	0.56	UK	europe	non-dryland	throughout	0.50	coarse	foulkes et al	2002
wheat	0.79	iran	asia	dryland	throughout	n/a	n/a	nouri et al	2011
wheat	0.98	USA	north america	dryland	throughout	0.74	coarse	english & nakamura	1989
wheat	0.96	USA	north america	dryland	throughout	0.63	coarse	english & nakamura	1989
wheat	0.69	USA	north america	dryland	throughout	0.39	coarse	english & nakamura	1989
wheat	0.50	USA	north america	dryland	throughout	0.27	coarse	english & nakamura	1989
wheat	0.99	USA	north america	dryland	throughout	0.85	coarse	english & nakamura	1989
wheat	0.93	USA	north america	dryland	throughout	0.73	coarse	english & nakamura	1989

wheat	0.63	USA	north america	dryland	throughout	0.43	coarse	english & nakamura	1989
wheat	0.48	USA	north america	dryland	throughout	0.26	coarse	english & nakamura	1989
wheat	0.95	USA	north america	dryland	throughout	0.77	coarse	english & nakamura	1989
wheat	0.92	USA	north america	dryland	throughout	0.78	coarse	english & nakamura	1989
wheat	0.79	USA	north america	dryland	throughout	0.82	coarse	english & nakamura	1989
wheat	0.77	USA	north america	dryland	throughout	0.60	coarse	english & nakamura	1989
wheat	0.95	USA	north america	dryland	throughout	0.65	coarse	english & nakamura	1989
wheat	1.01	pakistan	asia	dryland	throughout	0.75	medium	kahlown et al	2007
wheat	0.85	pakistan	asia	dryland	throughout	0.76	medium	kahlown et al	2007
maize	0.97	china	asia	dryland	throughout	0.88	medium	du et al	2010
maize	0.84	china	asia	dryland	throughout	0.75	medium	du et al	2010
maize	0.41	china	asia	dryland	throughout	0.63	medium	du et al	2010
maize	0.32	china	asia	dryland	throughout	0.50	medium	du et al	2010
maize	0.80	china	asia	dryland	throughout	0.86	medium	du et al	2010
maize	1.61	china	asia	dryland	throughout	0.60	medium	du et al	2010
maize	1.39	china	asia	dryland	throughout	0.51	medium	du et al	2010
maize	1.41	china	asia	dryland	throughout	0.40	medium	du et al	2010
maize	1.28	china	asia	dryland	throughout	0.34	medium	du et al	2010
wheat	0.95	china	asia	dryland	vegetative	n/a	medium	du et al	2010
wheat	0.93	china	asia	dryland	generative	n/a	medium	du et al	2011
wheat	0.90	china	asia	dryland	generative	n/a	medium	du et al	2012
wheat	0.82	china	asia	dryland	generative	n/a	medium	du et al	2013
wheat	0.80	iran	asia	dryland	throughout	0.63	n/a	zarea-fizabady & ghodzi	2004
wheat	0.60	iran	asia	dryland	throughout	0.50	n/a	zarea-fizabady & ghodzi	2004
wheat	0.96	pakistan	asia	dryland	vegetative	n/a	n/a	qadir et al	1999
wheat	0.91	pakistan	asia	dryland	generative	n/a	n/a	qadir et al	1999
wheat	0.90	pakistan	asia	dryland	generative	n/a	n/a	hussain et al	2004
wheat	0.83	pakistan	asia	dryland	generative	n/a	n/a	hussain et al	2004
wheat	0.71	pakistan	asia	dryland	throughout	n/a	n/a	hussain et al	2004
wheat	0.98	pakistan	asia	dryland	vegetative	n/a	n/a	hussain et al	2004
wheat	0.93	pakistan	asia	dryland	generative	n/a	n/a	hussain et al	2004
wheat	0.93	pakistan	asia	dryland	generative	n/a	n/a	hussain et al	2004
wheat	0.90	pakistan	asia	dryland	throughout	n/a	n/a	hussain et al	2004
wheat	1.03	pakistan	asia	dryland	vegetative	n/a	n/a	hussain et al	1997
wheat	1.09	pakistan	asia	dryland	generative	n/a	n/a	hussain et al	1997
wheat	0.93	pakistan	asia	dryland	throughout	n/a	n/a	hussain et al	1997
wheat	1.00	pakistan	asia	dryland	generative	n/a	n/a	hussain et al	1997
wheat	0.95	pakistan	asia	dryland	throughout	n/a	n/a	hussain et al	1997
wheat	0.74	iran	asia	dryland	generative	n/a	medium	johari-piretvalou	2010
wheat	1.12	china	asia	dryland	vegetative	0.75	coarse	li et al	2005
wheat	1.03	china	asia	dryland	vegetative	0.75	coarse	li et al	2005
wheat	0.94	china	asia	dryland	generative	0.50	coarse	li et al	2005
wheat	1.05	china	asia	dryland	generative	0.50	coarse	li et al	2005
wheat	0.95	china	asia	dryland	vegetative	0.50	coarse	li et al	2005
wheat	0.99	china	asia	dryland	generative	0.50	coarse	li et al	2005
wheat	0.96	china	asia	dryland	generative	0.25	coarse	li et al	2005
wheat	0.93	china	asia	dryland	generative	0.25	coarse	li et al	2005
wheat	0.92	china	asia	dryland	generative	0.25	coarse	li et al	2005
wheat	0.80	china	asia	dryland	throughout	0.29	coarse	li et al	2005
wheat	1.00	china	asia	dryland	generative	0.67	coarse	li et al	2005
wheat	0.88	china	asia	dryland	generative	0.33	coarse	li et al	2005
wheat	0.76	china	asia	dryland	throughout	0.24	coarse	li et al	2005
wheat	0.89	china	asia	dryland	generative	0.50	coarse	li et al	2005
wheat	0.92	china	asia	dryland	generative	0.50	coarse	li et al	2005
wheat	0.87	china	asia	dryland	generative	0.50	coarse	li et al	2005
wheat	0.74	china	asia	dryland	throughout	0.47	coarse	li et al	2005
wheat	0.97	banglades	asia	non-dryland	throughout	0.92	medium	ali et al	2007
wheat	0.88	banglades	asia	non-dryland	vegetative	0.74	medium	ali et al	2007
wheat	0.94	banglades	asia	non-dryland	vegetative	0.73	medium	ali et al	2007
wheat	0.92	banglades	asia	non-dryland	vegetative	0.71	medium	ali et al	2007
wheat	0.94	banglades	asia	non-dryland	generative	0.68	medium	ali et al	2007
wheat	0.84	banglades	asia	non-dryland	vegetative	0.59	medium	ali et al	2007
wheat	0.89	banglades	asia	non-dryland	generative	0.49	medium	ali et al	2007
wheat	0.80	banglades	asia	non-dryland	throughout	0.40	medium	ali et al	2007
wheat	0.49	banglades	asia	non-dryland	throughout	0.07	medium	ali et al	2007
wheat	0.84	USA	north america	dryland	generative	0.61	coarse	guttieri et al	1999
wheat	0.52	USA	north america	dryland	generative	0.22	coarse	guttieri et al	1999
wheat	0.16	australia	australia	dryland	throughout	0.09	fine	morgan & condon	1986
maize	0.89	turkey	asia	dryland	throughout	0.50	fine	kirda et al	2005
maize	0.80	china	asia	dryland	vegetative	0.98	medium	kang et al	2000
maize	0.61	china	asia	dryland	vegetative	0.75	medium	kang et al	2000
maize	0.80	china	asia	dryland	vegetative	0.81	medium	kang et al	2000
maize	0.99	china	asia	dryland	vegetative	0.77	medium	kang et al	2000
maize	0.65	china	asia	dryland	vegetative	0.79	medium	kang et al	2000
maize	0.86	china	asia	dryland	vegetative	0.96	medium	kang et al	2000
maize	0.92	china	asia	dryland	vegetative	0.76	medium	kang et al	2000
maize	0.62	china	asia	dryland	vegetative	0.70	medium	kang et al	2000
maize	0.87	china	asia	dryland	vegetative	0.95	medium	kang et al	2000
maize	0.66	china	asia	dryland	vegetative	0.72	medium	kang et al	2000
maize	0.86	china	asia	dryland	vegetative	0.79	medium	kang et al	2000

maize	0.99	china	asia	dryland	vegetative	0.81	medium	kang et al	2000
maize	0.69	china	asia	dryland	vegetative	0.76	medium	kang et al	2000
maize	0.85	china	asia	dryland	vegetative	0.87	medium	kang et al	2000
maize	0.92	china	asia	dryland	vegetative	0.71	medium	kang et al	2000
maize	0.68	china	asia	dryland	vegetative	0.67	medium	kang et al	2000
wheat	0.90	china	asia	dryland	throughout	0.63	coarse	pan et al	2003
wheat	0.65	china	asia	dryland	throughout	0.49	coarse	pan et al	2003
wheat	0.81	china	asia	dryland	throughout	0.61	coarse	pan et al	2003
wheat	0.53	china	asia	dryland	throughout	0.48	coarse	pan et al	2003
wheat	0.98	india	asia	non-dryland	throughout	0.99	coarse	panda et al	2003
wheat	0.95	india	asia	non-dryland	throughout	0.90	coarse	panda et al	2003
wheat	0.95	india	asia	non-dryland	throughout	0.85	coarse	panda et al	2003
wheat	0.95	india	asia	non-dryland	throughout	0.87	coarse	panda et al	2003
wheat	0.95	india	asia	non-dryland	throughout	0.97	coarse	panda et al	2003
wheat	0.93	india	asia	non-dryland	throughout	0.92	coarse	panda et al	2003
wheat	0.88	india	asia	non-dryland	throughout	0.88	coarse	panda et al	2003
wheat	0.86	india	asia	non-dryland	throughout	0.79	coarse	panda et al	2003
wheat	0.98	india	asia	non-dryland	throughout	0.89	coarse	panda et al	2003
wheat	0.95	india	asia	non-dryland	throughout	0.83	coarse	panda et al	2003
wheat	0.92	india	asia	non-dryland	throughout	0.82	coarse	panda et al	2003
wheat	0.92	india	asia	non-dryland	throughout	0.72	coarse	panda et al	2003
wheat	0.79	australia	australia	dryland	throughout	0.91	fine	steiner et al	1985
wheat	0.72	australia	australia	dryland	throughout	0.70	fine	steiner et al	1985
wheat	0.31	australia	australia	dryland	throughout	0.22	fine	steiner et al	1985
wheat	0.97	iran	asia	dryland	generative	0.89	medium	andarzian et al	2011
wheat	0.95	turkey	asia	dryland	throughout	0.77	medium	ilbeyi et al	2006
wheat	0.92	turkey	asia	dryland	throughout	0.53	medium	ilbeyi et al	2006
wheat	0.57	turkey	asia	dryland	throughout	0.30	medium	ilbeyi et al	2006
wheat	0.98	turkey	asia	dryland	throughout	0.78	medium	ilbeyi et al	2006
wheat	0.89	turkey	asia	dryland	throughout	0.56	medium	ilbeyi et al	2006
wheat	0.68	turkey	asia	dryland	throughout	0.33	medium	ilbeyi et al	2006
wheat	1.02	turkey	asia	dryland	throughout	0.84	medium	ilbeyi et al	2006
wheat	1.01	turkey	asia	dryland	throughout	0.68	medium	ilbeyi et al	2006
wheat	0.74	turkey	asia	dryland	throughout	0.52	medium	ilbeyi et al	2006
wheat	0.97	turkey	asia	dryland	throughout	0.85	medium	ilbeyi et al	2006
wheat	1.00	turkey	asia	dryland	throughout	0.70	medium	ilbeyi et al	2006
wheat	0.72	turkey	asia	dryland	throughout	0.55	medium	ilbeyi et al	2006
wheat	0.97	turkey	asia	dryland	throughout	0.78	medium	ilbeyi et al	2006
wheat	0.94	turkey	asia	dryland	throughout	0.55	medium	ilbeyi et al	2006
wheat	0.58	turkey	asia	dryland	throughout	0.33	medium	ilbeyi et al	2006
wheat	0.95	turkey	asia	dryland	throughout	0.79	medium	ilbeyi et al	2006
wheat	0.98	turkey	asia	dryland	throughout	0.58	medium	ilbeyi et al	2006
wheat	0.81	turkey	asia	dryland	throughout	0.37	medium	ilbeyi et al	2006
wheat	0.97	turkey	asia	dryland	throughout	0.82	medium	ilbeyi et al	2006
wheat	0.90	turkey	asia	dryland	throughout	0.64	medium	ilbeyi et al	2006
wheat	0.71	turkey	asia	dryland	throughout	0.46	medium	ilbeyi et al	2006
wheat	0.96	turkey	asia	dryland	throughout	0.83	medium	ilbeyi et al	2006
wheat	0.95	turkey	asia	dryland	throughout	0.66	medium	ilbeyi et al	2006
wheat	0.86	turkey	asia	dryland	throughout	0.49	medium	ilbeyi et al	2006
wheat	0.93	USA	north america	dryland	throughout	0.87	coarse	miller & hang	1982
wheat	0.84	USA	north america	dryland	throughout	0.73	coarse	miller & hang	1982
wheat	0.70	USA	north america	dryland	throughout	0.60	coarse	miller & hang	1982
wheat	0.52	USA	north america	dryland	throughout	0.47	coarse	miller & hang	1982
wheat	0.36	USA	north america	dryland	throughout	0.33	coarse	miller & hang	1982
wheat	0.16	USA	north america	dryland	throughout	0.20	coarse	miller & hang	1982
wheat	0.92	USA	north america	dryland	throughout	0.85	coarse	miller & hang	1982
wheat	0.69	USA	north america	dryland	throughout	0.69	coarse	miller & hang	1982
wheat	0.79	USA	north america	dryland	throughout	0.54	coarse	miller & hang	1982
wheat	0.73	USA	north america	dryland	throughout	0.38	coarse	miller & hang	1982
wheat	0.59	USA	north america	dryland	throughout	0.23	coarse	miller & hang	1982
wheat	1.04	USA	north america	dryland	throughout	0.90	medium	miller & hang	1982
wheat	1.06	USA	north america	dryland	throughout	0.81	medium	miller & hang	1982
wheat	1.24	USA	north america	dryland	throughout	0.71	medium	miller & hang	1982
wheat	1.06	USA	north america	dryland	throughout	0.62	medium	miller & hang	1982
wheat	1.22	USA	north america	dryland	throughout	0.52	medium	miller & hang	1982
wheat	1.10	USA	north america	dryland	throughout	0.43	medium	miller & hang	1982
wheat	0.96	USA	north america	dryland	throughout	0.33	medium	miller & hang	1982
wheat	0.84	USA	north america	dryland	throughout	0.24	medium	miller & hang	1982
wheat	0.52	USA	north america	dryland	throughout	0.14	medium	miller & hang	1982
wheat	0.96	USA	north america	dryland	throughout	0.87	medium	miller & hang	1982
wheat	0.95	USA	north america	dryland	throughout	0.73	medium	miller & hang	1982
wheat	0.95	USA	north america	dryland	throughout	0.60	medium	miller & hang	1982
wheat	0.95	USA	north america	dryland	throughout	0.47	medium	miller & hang	1982
wheat	0.96	USA	north america	dryland	throughout	0.33	medium	miller & hang	1982
wheat	0.82	USA	north america	dryland	throughout	0.20	medium	miller & hang	1982
maize	0.81	tanzania	africa	non-dryland	vegetative	0.84	medium	igbadun et al	2006
maize	0.73	tanzania	africa	non-dryland	generative	0.86	medium	igbadun et al	2006
maize	0.74	tanzania	africa	non-dryland	generative	0.87	medium	igbadun et al	2006
maize	0.60	tanzania	africa	non-dryland	generative	0.70	medium	igbadun et al	2006
maize	0.72	tanzania	africa	non-dryland	generative	0.71	medium	igbadun et al	2006

maize	0.60	tanzania	africa	non-dryland	generative	0.73	medium	igbadun et al	2006
maize	0.43	tanzania	africa	non-dryland	generative	0.57	medium	igbadun et al	2006
maize	0.95	tanzania	africa	non-dryland	vegetative	0.84	medium	igbadun et al	2006
maize	0.71	tanzania	africa	non-dryland	generative	0.86	medium	igbadun et al	2006
maize	0.80	tanzania	africa	non-dryland	generative	0.87	medium	igbadun et al	2006
maize	0.69	tanzania	africa	non-dryland	generative	0.70	medium	igbadun et al	2006
maize	0.81	tanzania	africa	non-dryland	generative	0.71	medium	igbadun et al	2006
maize	0.73	tanzania	africa	non-dryland	generative	0.73	medium	igbadun et al	2006
maize	0.53	tanzania	africa	non-dryland	generative	0.57	medium	igbadun et al	2006
maize	0.84	tanzania	africa	non-dryland	vegetative	0.84	medium	igbadun et al	2006
maize	0.78	tanzania	africa	non-dryland	generative	0.86	medium	igbadun et al	2006
maize	0.75	tanzania	africa	non-dryland	generative	0.87	medium	igbadun et al	2006
maize	0.44	tanzania	africa	non-dryland	generative	0.57	medium	igbadun et al	2006
wheat	0.44	australia	australia	dryland	generative	n/a	medium	thomson & chase	1992
wheat	0.80	australia	australia	dryland	generative	n/a	medium	thomson & chase	1992
wheat	0.47	australia	australia	dryland	vegetative	n/a	medium	thomson & chase	1992
wheat	0.36	australia	australia	dryland	generative	n/a	medium	thomson & chase	1992
wheat	0.14	australia	australia	dryland	throughout	n/a	medium	thomson & chase	1992
wheat	0.90	india	asia	dryland	generative	0.84	coarse	sharma et al	1990
wheat	0.93	india	asia	dryland	generative	0.84	coarse	sharma et al	1990
wheat	0.71	india	asia	dryland	generative	0.68	coarse	sharma et al	1990
wheat	0.84	india	asia	dryland	generative	0.68	coarse	sharma et al	1990
wheat	0.73	india	asia	dryland	vegetative	0.68	coarse	sharma et al	1990
wheat	0.58	india	asia	dryland	generative	0.52	coarse	sharma et al	1990
wheat	0.40	india	asia	dryland	throughout	0.36	coarse	sharma et al	1990
wheat	0.15	india	asia	dryland	throughout	0.20	coarse	sharma et al	1990
wheat	0.83	india	asia	dryland	generative	0.84	coarse	sharma et al	1990
wheat	0.96	india	asia	dryland	generative	0.84	coarse	sharma et al	1990
wheat	0.79	india	asia	dryland	generative	0.69	coarse	sharma et al	1990
wheat	0.90	india	asia	dryland	generative	0.69	coarse	sharma et al	1990
wheat	0.69	india	asia	dryland	vegetative	0.69	coarse	sharma et al	1990
wheat	0.57	india	asia	dryland	generative	0.53	coarse	sharma et al	1990
wheat	0.34	india	asia	dryland	throughout	0.48	coarse	sharma et al	1990
wheat	0.21	india	asia	dryland	throughout	0.22	coarse	sharma et al	1990
wheat	1.01	china	asia	dryland	throughout	0.83	medium	liu et al	2011
wheat	1.01	china	asia	dryland	throughout	0.67	medium	liu et al	2011
wheat	1.07	china	asia	dryland	throughout	0.80	medium	liu et al	2011
wheat	0.93	china	asia	dryland	throughout	0.63	medium	liu et al	2011
wheat	0.78	china	asia	dryland	throughout	0.46	medium	liu et al	2011
wheat	1.04	china	asia	dryland	throughout	0.95	medium	liu et al	2011
wheat	0.95	china	asia	dryland	throughout	0.91	medium	liu et al	2011
wheat	0.98	china	asia	dryland	throughout	0.86	medium	liu et al	2011
wheat	0.46	india	asia	non-dryland	throughout	0.34	coarse	innes & blackwell	1981
wheat	0.56	india	asia	non-dryland	generative	0.53	coarse	innes & blackwell	1981
wheat	0.57	india	asia	non-dryland	vegetative	0.79	coarse	innes & blackwell	1981
wheat	0.52	india	asia	non-dryland	vegetative	0.87	coarse	innes & blackwell	1981
wheat	0.27	india	asia	non-dryland	generative	0.39	coarse	innes & blackwell	1981
wheat	1.02	india	asia	non-dryland	generative	0.88	medium	mishra et al	1999
wheat	1.02	india	asia	non-dryland	generative	0.76	medium	mishra et al	1999
wheat	1.01	india	asia	non-dryland	vegetative	0.64	medium	mishra et al	1999
wheat	1.00	india	asia	non-dryland	throughout	0.52	medium	mishra et al	1999
wheat	0.68	india	asia	non-dryland	throughout	0.40	medium	mishra et al	1999
wheat	1.02	india	asia	non-dryland	generative	0.83	medium	mishra et al	1999
wheat	1.03	india	asia	non-dryland	generative	0.66	medium	mishra et al	1999
wheat	1.02	india	asia	non-dryland	vegetative	0.49	medium	mishra et al	1999
wheat	0.64	india	asia	non-dryland	throughout	0.32	medium	mishra et al	1999
wheat	0.57	india	asia	non-dryland	throughout	0.15	medium	mishra et al	1999
wheat	1.03	india	asia	non-dryland	generative	0.88	medium	mishra et al	1999
wheat	1.03	india	asia	non-dryland	generative	0.76	medium	mishra et al	1999
wheat	0.98	india	asia	non-dryland	vegetative	0.64	medium	mishra et al	1999
wheat	0.96	india	asia	non-dryland	throughout	0.52	medium	mishra et al	1999
wheat	0.67	india	asia	non-dryland	throughout	0.40	medium	mishra et al	1999
wheat	1.03	india	asia	non-dryland	generative	0.83	medium	mishra et al	1999
wheat	1.01	india	asia	non-dryland	generative	0.66	medium	mishra et al	1999
wheat	0.99	india	asia	non-dryland	vegetative	0.49	medium	mishra et al	1999
wheat	0.60	india	asia	non-dryland	throughout	0.32	medium	mishra et al	1999
wheat	0.54	india	asia	non-dryland	throughout	0.15	medium	mishra et al	1999
wheat	0.94	england	europe	non-dryland	generative	0.11	medium	gales & wilson	1981
wheat	0.97	england	europe	non-dryland	generative	0.15	medium	gales & wilson	1981
wheat	1.02	england	europe	non-dryland	generative	0.09	medium	gales & wilson	1981
wheat	0.92	england	europe	non-dryland	generative	0.11	medium	gales & wilson	1981
wheat	0.95	england	europe	non-dryland	generative	0.13	medium	gales & wilson	1981
wheat	0.91	england	europe	non-dryland	generative	0.09	medium	gales & wilson	1981
wheat	0.98	england	europe	non-dryland	generative	0.12	medium	gales & wilson	1981
wheat	1.02	england	europe	non-dryland	generative	0.16	medium	gales & wilson	1981
wheat	0.96	england	europe	non-dryland	generative	0.14	medium	gales & wilson	1981
wheat	0.99	england	europe	non-dryland	generative	0.09	medium	gales & wilson	1981
wheat	1.07	england	europe	non-dryland	generative	0.06	medium	gales & wilson	1981
wheat	0.97	england	europe	non-dryland	generative	0.07	medium	gales & wilson	1981
wheat	0.99	banglades	asia	non-dryland	vegetative	0.79	medium	rahman et al	1981

wheat	0.81	banglades	asia	non-dryland	generative	0.62	medium	rahman et al	1981
wheat	1.09	banglades	asia	non-dryland	generative	0.75	medium	rahman et al	1981
wheat	0.52	banglades	asia	non-dryland	vegetative	0.46	medium	rahman et al	1981
wheat	0.80	banglades	asia	non-dryland	generative	0.37	medium	rahman et al	1981
wheat	0.69	banglades	asia	non-dryland	generative	0.25	medium	rahman et al	1981
wheat	0.57	banglades	asia	non-dryland	throughout	0.01	medium	rahman et al	1981
wheat	0.58	USA	north america	dryland	vegetative	0.87	coarse	ehdaie	1995
wheat	0.42	denmark	europe	non-dryland	vegetative	0.35	coarse	mogensen et al	1985
wheat	0.50	denmark	europe	non-dryland	vegetative	0.20	coarse	mogensen et al	1985
wheat	0.51	denmark	europe	non-dryland	vegetative	0.20	coarse	mogensen et al	1985
wheat	0.63	denmark	europe	non-dryland	generative	0.11	coarse	mogensen et al	1985
wheat	0.81	denmark	europe	non-dryland	generative	0.07	coarse	mogensen et al	1985
wheat	0.88	denmark	europe	non-dryland	generative	0.05	coarse	mogensen et al	1985
wheat	0.85	denmark	europe	non-dryland	generative	0.16	coarse	mogensen et al	1985
wheat	0.83	denmark	europe	non-dryland	vegetative	0.35	coarse	mogensen et al	1985
wheat	0.84	denmark	europe	non-dryland	vegetative	0.20	coarse	mogensen et al	1985
wheat	0.69	denmark	europe	non-dryland	vegetative	0.20	coarse	mogensen et al	1985
wheat	0.63	denmark	europe	non-dryland	generative	0.11	coarse	mogensen et al	1985
wheat	0.81	denmark	europe	non-dryland	generative	0.07	coarse	mogensen et al	1985
wheat	0.88	denmark	europe	non-dryland	generative	0.05	coarse	mogensen et al	1985
wheat	0.85	denmark	europe	non-dryland	generative	0.16	coarse	mogensen et al	1985
wheat	0.76	germany	europe	non-dryland	vegetative	n/a	coarse	singh et al	1987
wheat	0.81	germany	europe	non-dryland	generative	n/a	coarse	singh et al	1987
wheat	0.99	germany	europe	non-dryland	generative	n/a	coarse	singh et al	1987
wheat	0.81	germany	europe	non-dryland	vegetative	n/a	coarse	singh et al	1987
wheat	0.65	germany	europe	non-dryland	generative	n/a	coarse	singh et al	1987
wheat	1.12	germany	europe	non-dryland	generative	n/a	coarse	singh et al	1987
wheat	0.37	germany	europe	non-dryland	throughout	n/a	coarse	singh et al	1987
wheat	0.87	india	asia	dryland	throughout	n/a	n/a	singh et al	1987
wheat	0.82	india	asia	dryland	vegetative	n/a	n/a	singh et al	1987
wheat	0.83	india	asia	dryland	vegetative	n/a	n/a	singh et al	1987
wheat	0.99	india	asia	non-dryland	throughout	n/a	n/a	chaturvedi et al	1981
wheat	0.53	india	asia	non-dryland	throughout	n/a	n/a	chaturvedi et al	1981
maize	0.94	USA	north america	dryland	throughout	0.91	medium	retta & hanks	1980
maize	0.84	USA	north america	dryland	throughout	0.72	medium	retta & hanks	1980
maize	0.74	USA	north america	dryland	throughout	0.59	medium	retta & hanks	1980
maize	0.53	USA	north america	dryland	throughout	0.39	medium	retta & hanks	1980
maize	0.37	USA	north america	dryland	throughout	0.30	medium	retta & hanks	1980
maize	0.88	USA	north america	dryland	generative	0.87	medium	retta & hanks	1980
maize	0.93	USA	north america	dryland	generative	0.79	medium	retta & hanks	1980
maize	0.75	USA	north america	dryland	generative	0.60	medium	retta & hanks	1980
maize	0.47	USA	north america	dryland	generative	0.46	medium	retta & hanks	1980
maize	0.32	USA	north america	dryland	generative	0.41	medium	retta & hanks	1980
maize	0.82	USA	north america	dryland	vegetative	0.94	medium	retta & hanks	1980
maize	0.96	USA	north america	dryland	vegetative	0.86	medium	retta & hanks	1980
maize	0.97	USA	north america	dryland	vegetative	0.75	medium	retta & hanks	1980
maize	0.92	USA	north america	dryland	vegetative	0.64	medium	retta & hanks	1980
maize	0.74	USA	north america	dryland	vegetative	0.62	medium	retta & hanks	1980
wheat	0.76	iran	asia	dryland	generative	0.82	medium	pireivatlou et al	2010
wheat	1.11	iran	asia	dryland	throughout	0.97	n/a	salemi et al	2011
wheat	0.89	iran	asia	dryland	throughout	0.89	n/a	salemi et al	2011
wheat	0.92	iran	asia	dryland	throughout	0.77	n/a	salemi et al	2011
wheat	0.97	iran	asia	dryland	throughout	0.76	n/a	salemi et al	2011
wheat	0.80	iran	asia	dryland	throughout	0.68	n/a	salemi et al	2011
wheat	0.93	iran	asia	dryland	throughout	0.67	n/a	salemi et al	2011
wheat	0.83	iran	asia	dryland	throughout	0.64	n/a	salemi et al	2011
wheat	0.69	iran	asia	dryland	throughout	0.61	n/a	salemi et al	2011
wheat	0.79	USA	north america	dryland	generative	0.64	medium	johnson & kanemasu	1982
wheat	0.72	USA	north america	dryland	vegetative	0.61	medium	johnson & kanemasu	1982
wheat	1.07	USA	north america	dryland	generative	0.38	medium	johnson & kanemasu	1982
wheat	0.74	USA	north america	dryland	vegetative	0.38	medium	johnson & kanemasu	1982
wheat	0.63	USA	north america	dryland	throughout	0.33	medium	johnson & kanemasu	1982
wheat	1.03	china	asia	dryland	generative	0.92	coarse	liao et al	2008
wheat	1.00	china	asia	dryland	generative	0.85	coarse	liao et al	2008
wheat	0.96	china	asia	dryland	generative	0.77	coarse	liao et al	2008
wheat	0.86	china	asia	dryland	generative	0.69	coarse	liao et al	2008
wheat	0.81	china	asia	dryland	generative	0.62	coarse	liao et al	2008
wheat	0.74	china	asia	dryland	generative	0.54	coarse	liao et al	2008
wheat	0.64	china	asia	dryland	throughout	0.46	coarse	liao et al	2008
wheat	0.54	china	asia	dryland	throughout	0.38	coarse	liao et al	2008
wheat	0.43	china	asia	dryland	throughout	0.31	coarse	liao et al	2008
wheat	1.03	china	asia	dryland	generative	0.92	coarse	liao et al	2008
wheat	0.99	china	asia	dryland	generative	0.85	coarse	liao et al	2008
wheat	0.96	china	asia	dryland	generative	0.77	coarse	liao et al	2008
wheat	0.85	china	asia	dryland	generative	0.69	coarse	liao et al	2008
wheat	0.79	china	asia	dryland	generative	0.62	coarse	liao et al	2008
wheat	0.74	china	asia	dryland	generative	0.54	coarse	liao et al	2008
wheat	0.29	india	asia	dryland	throughout	0.22	n/a	singh et al	2012
maize	0.94	thailand	asia	non-dryland	throughout	0.33	medium	weerathaworn et al	1992
maize	0.77	thailand	asia	non-dryland	generative	0.67	medium	weerathaworn et al	1992

maize	0.92	thailand	asia	non-dryland	vegetative	0.67	medium	weerathaworn et al	1992
maize	0.78	thailand	asia	non-dryland	throughout	0.17	medium	weerathaworn et al	1992
maize	0.88	thailand	asia	non-dryland	generative	0.58	medium	weerathaworn et al	1992
maize	0.86	thailand	asia	non-dryland	vegetative	0.58	medium	weerathaworn et al	1992
wheat	0.61	turkey	asia	dryland	throughout	0.81	medium	ozturk & aydin	2004
wheat	0.60	turkey	asia	dryland	vegetative	0.81	medium	ozturk & aydin	2004
wheat	0.78	turkey	asia	dryland	generative	0.88	medium	ozturk & aydin	2004
wheat	0.33	turkey	asia	dryland	throughout	0.65	medium	ozturk & aydin	2004
wheat	0.77	turkey	asia	dryland	throughout	0.87	medium	ozturk & aydin	2004
wheat	0.59	turkey	asia	dryland	vegetative	0.84	medium	ozturk & aydin	2004
wheat	0.74	turkey	asia	dryland	generative	0.91	medium	ozturk & aydin	2004
wheat	0.36	turkey	asia	dryland	throughout	0.71	medium	ozturk & aydin	2004
wheat	0.93	china	asia	dryland	generative	0.86	medium	huang et al	2004
wheat	0.86	china	asia	dryland	generative	0.72	medium	huang et al	2004
wheat	0.78	china	asia	dryland	generative	0.58	medium	huang et al	2004
wheat	0.65	china	asia	dryland	throughout	0.44	medium	huang et al	2004
maize	0.80	china	asia	dryland	throughout	0.74	medium	liu et al	2010
maize	0.73	china	asia	dryland	throughout	0.82	medium	liu et al	2010
maize	0.88	USA	north america	dryland	vegetative	0.89	medium	payero et al	2006
maize	0.91	USA	north america	dryland	generative	0.83	medium	payero et al	2006
maize	0.82	USA	north america	dryland	generative	0.93	medium	payero et al	2006
maize	0.84	USA	north america	dryland	generative	0.77	medium	payero et al	2006
maize	0.78	USA	north america	dryland	throughout	0.63	medium	payero et al	2006
maize	0.72	USA	north america	dryland	throughout	0.72	medium	payero et al	2006
maize	0.24	USA	north america	dryland	throughout	0.26	medium	payero et al	2006
maize	0.99	USA	north america	dryland	vegetative	0.83	medium	payero et al	2006
maize	0.87	USA	north america	dryland	throughout	0.79	medium	payero et al	2006
maize	0.93	USA	north america	dryland	vegetative	0.87	medium	payero et al	2006
maize	1.00	USA	north america	dryland	throughout	0.83	medium	payero et al	2006
maize	0.87	USA	north america	dryland	throughout	0.80	medium	payero et al	2006
maize	0.90	USA	north america	dryland	throughout	0.78	medium	payero et al	2006
maize	0.81	USA	north america	dryland	throughout	0.78	medium	payero et al	2006
maize	0.56	USA	north america	dryland	throughout	0.72	medium	payero et al	2006
wheat	0.95	mexico	south america	dryland	vegetative	0.80	coarse	moinuddin et al	2005
wheat	0.77	mexico	south america	dryland	vegetative	0.60	coarse	moinuddin et al	2005
wheat	0.53	mexico	south america	dryland	vegetative	0.42	coarse	moinuddin et al	2005
wheat	0.34	mexico	south america	dryland	vegetative	0.28	coarse	moinuddin et al	2005
wheat	1.13	mexico	south america	dryland	vegetative	0.86	coarse	moinuddin et al	2005
wheat	1.05	mexico	south america	dryland	vegetative	0.86	coarse	moinuddin et al	2005
wheat	0.71	mexico	south america	dryland	vegetative	0.80	coarse	moinuddin et al	2005
wheat	0.63	mexico	south america	dryland	generative	0.59	coarse	moinuddin et al	2005
wheat	0.34	mexico	south america	dryland	generative	0.45	coarse	moinuddin et al	2005
wheat	0.96	mexico	south america	dryland	throughout	0.91	coarse	moinuddin et al	2005
wheat	0.82	mexico	south america	dryland	throughout	0.66	coarse	moinuddin et al	2005
wheat	0.69	mexico	south america	dryland	throughout	0.49	coarse	moinuddin et al	2005
wheat	0.57	mexico	south america	dryland	throughout	0.41	coarse	moinuddin et al	2005
wheat	0.95	mexico	south america	dryland	throughout	0.91	coarse	moinuddin et al	2005
wheat	0.81	mexico	south america	dryland	throughout	0.66	coarse	moinuddin et al	2005
wheat	0.67	mexico	south america	dryland	throughout	0.49	coarse	moinuddin et al	2005
wheat	0.53	mexico	south america	dryland	throughout	0.41	coarse	moinuddin et al	2005
wheat	1.10	china	asia	non-dryland	generative	0.85	medium	zhang et al	2008
wheat	1.05	china	asia	non-dryland	generative	0.70	medium	zhang et al	2008
wheat	1.04	china	asia	non-dryland	generative	0.54	medium	zhang et al	2008
wheat	0.84	china	asia	non-dryland	throughout	0.39	medium	zhang et al	2008
wheat	0.79	china	asia	non-dryland	throughout	0.22	medium	zhang et al	2008
wheat	1.02	china	asia	non-dryland	generative	0.86	medium	zhang et al	2008
wheat	1.18	china	asia	non-dryland	generative	0.73	medium	zhang et al	2008
wheat	1.20	china	asia	non-dryland	generative	0.73	medium	zhang et al	2008
wheat	1.13	china	asia	non-dryland	throughout	0.45	medium	zhang et al	2008
wheat	0.79	china	asia	non-dryland	throughout	0.29	medium	zhang et al	2008
wheat	1.02	china	asia	non-dryland	generative	0.88	medium	zhang et al	2008
wheat	1.02	china	asia	non-dryland	generative	0.75	medium	zhang et al	2008
wheat	1.00	china	asia	non-dryland	generative	0.63	medium	zhang et al	2008
wheat	1.03	china	asia	non-dryland	throughout	0.50	medium	zhang et al	2008
wheat	1.08	china	asia	non-dryland	throughout	0.36	medium	zhang et al	2008
wheat	1.35	china	asia	non-dryland	generative	0.89	medium	zhang et al	2008
wheat	1.33	china	asia	non-dryland	generative	0.77	medium	zhang et al	2008
wheat	1.38	china	asia	non-dryland	generative	0.66	medium	zhang et al	2008
wheat	1.39	china	asia	non-dryland	throughout	0.54	medium	zhang et al	2008
wheat	1.29	china	asia	non-dryland	throughout	0.41	medium	zhang et al	2008
wheat	1.39	china	asia	non-dryland	generative	0.86	medium	zhang et al	2008
wheat	1.38	china	asia	non-dryland	generative	0.72	medium	zhang et al	2008
wheat	1.44	china	asia	non-dryland	generative	0.58	medium	zhang et al	2008
wheat	1.39	china	asia	non-dryland	throughout	0.44	medium	zhang et al	2008
wheat	1.21	china	asia	non-dryland	throughout	0.27	medium	zhang et al	2008
wheat	1.11	china	asia	non-dryland	generative	0.85	medium	zhang et al	2008
wheat	1.13	china	asia	non-dryland	generative	0.69	medium	zhang et al	2008
wheat	1.05	china	asia	non-dryland	generative	0.54	medium	zhang et al	2008
wheat	0.87	china	asia	non-dryland	throughout	0.39	medium	zhang et al	2008
wheat	0.27	china	asia	non-dryland	throughout	0.21	medium	zhang et al	2008

maize	0.78	USA	north america	non-dryland	vegetative	0.93	coarse	heng et al	2009
maize	0.26	USA	north america	non-dryland	throughout	0.72	coarse	heng et al	2009
maize	1.00	spain	europe	dryland	throughout	0.57	medium	heng et al	2009
maize	0.82	spain	europe	dryland	throughout	0.31	medium	heng et al	2009
wheat	0.97	china	asia	dryland	vegetative	n/a	medium	zhang et al	2005
wheat	1.00	china	asia	dryland	generative	n/a	medium	zhang et al	2005
wheat	0.99	china	asia	dryland	generative	n/a	medium	zhang et al	2005
wheat	1.00	china	asia	dryland	generative	n/a	medium	zhang et al	2005
wheat	0.99	china	asia	dryland	generative	n/a	medium	zhang et al	2005
wheat	0.96	china	asia	dryland	throughout	n/a	medium	zhang et al	2005
wheat	1.02	china	asia	dryland	vegetative	n/a	medium	zhang et al	2005
wheat	1.00	china	asia	dryland	generative	n/a	medium	zhang et al	2005
wheat	1.02	china	asia	dryland	generative	n/a	medium	zhang et al	2005
wheat	1.03	china	asia	dryland	generative	n/a	medium	zhang et al	2005
wheat	1.00	china	asia	dryland	generative	n/a	medium	zhang et al	2005
wheat	1.02	china	asia	dryland	throughout	n/a	medium	zhang et al	2005
wheat	1.05	china	asia	dryland	vegetative	n/a	medium	zhang et al	2005
wheat	1.03	china	asia	dryland	generative	n/a	medium	zhang et al	2005
wheat	1.05	china	asia	dryland	generative	n/a	medium	zhang et al	2005
wheat	1.03	china	asia	dryland	generative	n/a	medium	zhang et al	2005
wheat	1.03	china	asia	dryland	generative	n/a	medium	zhang et al	2005
wheat	0.95	china	asia	dryland	throughout	n/a	medium	zhang et al	2005
wheat	1.03	china	asia	dryland	vegetative	n/a	medium	zhang et al	2005
wheat	0.99	china	asia	dryland	generative	n/a	medium	zhang et al	2005
wheat	1.00	china	asia	dryland	generative	n/a	medium	zhang et al	2005
wheat	0.79	china	asia	dryland	generative	n/a	medium	zhang et al	2005
wheat	0.85	china	asia	dryland	generative	n/a	medium	zhang et al	2005
wheat	0.75	china	asia	dryland	throughout	n/a	medium	zhang et al	2005
wheat	1.00	china	asia	dryland	vegetative	n/a	medium	zhang et al	2005
wheat	0.91	china	asia	dryland	generative	n/a	medium	zhang et al	2005
wheat	0.93	china	asia	dryland	generative	n/a	medium	zhang et al	2005
wheat	0.91	china	asia	dryland	generative	n/a	medium	zhang et al	2005
wheat	0.88	china	asia	dryland	generative	n/a	medium	zhang et al	2005
wheat	0.76	china	asia	dryland	throughout	n/a	medium	zhang et al	2005
wheat	1.01	china	asia	dryland	generative	0.73	medium	hu et al	2005
wheat	0.83	china	asia	dryland	generative	0.47	medium	hu et al	2005
wheat	0.77	china	asia	dryland	throughout	0.20	medium	hu et al	2005
wheat	0.93	china	asia	dryland	generative	0.73	medium	hu et al	2005
wheat	0.80	china	asia	dryland	generative	0.46	medium	hu et al	2005
wheat	0.66	china	asia	dryland	throughout	0.18	medium	hu et al	2005
wheat	0.94	china	asia	dryland	generative	0.75	medium	hu et al	2005
wheat	0.77	china	asia	dryland	generative	0.51	medium	hu et al	2005
wheat	0.72	china	asia	dryland	throughout	0.26	medium	hu et al	2005
wheat	1.18	china	asia	dryland	generative	0.77	medium	hu et al	2005
wheat	1.10	china	asia	dryland	generative	0.53	medium	hu et al	2005
wheat	0.77	china	asia	dryland	throughout	0.30	medium	hu et al	2005
maize	0.85	tanzania	africa	non-dryland	vegetative	0.84	coarse	igbadun et al	2008
maize	0.74	tanzania	africa	non-dryland	generative	0.86	coarse	igbadun et al	2008
maize	0.78	tanzania	africa	non-dryland	generative	0.87	coarse	igbadun et al	2008
maize	0.62	tanzania	africa	non-dryland	generative	0.70	coarse	igbadun et al	2008
maize	0.72	tanzania	africa	non-dryland	generative	0.71	coarse	igbadun et al	2008
maize	0.61	tanzania	africa	non-dryland	generative	0.73	coarse	igbadun et al	2008
maize	0.45	tanzania	africa	non-dryland	throughout	0.57	coarse	igbadun et al	2008
maize	0.88	tanzania	africa	non-dryland	vegetative	0.83	coarse	igbadun et al	2008
maize	0.75	tanzania	africa	non-dryland	generative	0.87	coarse	igbadun et al	2008
maize	0.77	tanzania	africa	non-dryland	generative	0.89	coarse	igbadun et al	2008
maize	0.57	tanzania	africa	non-dryland	generative	0.69	coarse	igbadun et al	2008
maize	0.65	tanzania	africa	non-dryland	generative	0.72	coarse	igbadun et al	2008
maize	0.56	tanzania	africa	non-dryland	generative	0.76	coarse	igbadun et al	2008
maize	0.37	tanzania	africa	non-dryland	throughout	0.59	coarse	igbadun et al	2008
wheat	0.61	USA	north america	dryland	generative	n/a	n/a	butler et al	2005
maize	1.05	india	asia	dryland	vegetative	0.85	medium	mishra et al	2001
maize	1.07	india	asia	dryland	generative	0.70	medium	mishra et al	2001
maize	1.03	india	asia	dryland	generative	0.80	medium	mishra et al	2001
maize	0.88	india	asia	dryland	generative	0.40	medium	mishra et al	2001
maize	0.64	india	asia	dryland	throughout	0.10	medium	mishra et al	2001
maize	1.00	india	asia	dryland	vegetative	0.85	medium	mishra et al	2001
maize	1.03	india	asia	dryland	generative	0.71	medium	mishra et al	2001
maize	0.99	india	asia	dryland	generative	0.56	medium	mishra et al	2001
maize	0.85	india	asia	dryland	generative	0.42	medium	mishra et al	2001
maize	0.58	india	asia	dryland	throughout	0.13	medium	mishra et al	2001
wheat	0.88	china	asia	dryland	vegetative	0.94	medium	zhang et al	1999
wheat	0.86	china	asia	dryland	vegetative	0.90	medium	zhang et al	1999
wheat	1.07	china	asia	dryland	generative	0.88	medium	zhang et al	1999
wheat	1.06	china	asia	dryland	generative	0.72	medium	zhang et al	1999
wheat	1.00	china	asia	dryland	generative	0.60	medium	zhang et al	1999
wheat	0.73	china	asia	dryland	throughout	0.40	medium	zhang et al	1999
wheat	0.52	china	asia	dryland	throughout	0.25	medium	zhang et al	1999
wheat	1.11	china	asia	dryland	vegetative	0.97	medium	zhang et al	1999
wheat	1.10	china	asia	dryland	vegetative	0.82	medium	zhang et al	1999

wheat	1.15	china	asia	dryland	generative	0.72	medium	zhang et al	1999
wheat	1.12	china	asia	dryland	generative	0.60	medium	zhang et al	1999
wheat	1.05	china	asia	dryland	generative	0.45	medium	zhang et al	1999
wheat	0.84	china	asia	dryland	throughout	0.28	medium	zhang et al	1999
wheat	0.59	china	asia	dryland	throughout	0.20	medium	zhang et al	1999
wheat	1.06	china	asia	dryland	vegetative	0.90	coarse	zhang et al	1999
wheat	1.10	china	asia	dryland	generative	0.79	coarse	zhang et al	1999
wheat	1.10	china	asia	dryland	generative	0.68	coarse	zhang et al	1999
wheat	1.00	china	asia	dryland	throughout	0.49	coarse	zhang et al	1999
wheat	0.67	china	asia	dryland	throughout	0.32	coarse	zhang et al	1999
wheat	0.92	china	asia	dryland	vegetative	0.90	medium	zhang et al	1999
wheat	0.96	china	asia	dryland	generative	0.79	medium	zhang et al	1999
wheat	0.79	china	asia	dryland	generative	0.65	medium	zhang et al	1999
wheat	0.58	china	asia	dryland	throughout	0.49	medium	zhang et al	1999
wheat	0.54	china	asia	dryland	throughout	0.31	medium	zhang et al	1999
wheat	1.05	china	asia	dryland	generative	0.66	coarse	sun et al	2006
wheat	1.03	china	asia	dryland	vegetative	0.82	coarse	sun et al	2006
wheat	1.03	china	asia	dryland	vegetative	0.74	coarse	sun et al	2006
wheat	0.67	china	asia	dryland	throughout	0.29	coarse	sun et al	2006
wheat	1.04	china	asia	dryland	generative	0.82	coarse	sun et al	2006
wheat	1.00	china	asia	dryland	vegetative	0.85	coarse	sun et al	2006
wheat	0.98	china	asia	dryland	vegetative	0.92	coarse	sun et al	2006
wheat	0.67	china	asia	dryland	throughout	0.49	coarse	sun et al	2006
wheat	1.02	china	asia	dryland	generative	0.78	coarse	sun et al	2006
wheat	1.02	china	asia	dryland	vegetative	0.77	coarse	sun et al	2006
wheat	0.99	china	asia	dryland	vegetative	0.77	coarse	sun et al	2006
wheat	0.81	china	asia	dryland	throughout	0.41	coarse	sun et al	2006
wheat	0.97	china	asia	dryland	generative	0.68	n/a	han et al	2008
maize	0.93	USA	north america	non-dryland	throughout	0.48	coarse	steele et al	1994
maize	0.97	USA	north america	non-dryland	throughout	0.65	coarse	steele et al	1994
maize	0.96	USA	north america	non-dryland	throughout	0.50	coarse	steele et al	1994
maize	0.98	USA	north america	non-dryland	throughout	0.49	coarse	steele et al	1994
maize	0.96	USA	north america	non-dryland	throughout	0.44	coarse	steele et al	1994
maize	0.96	USA	north america	non-dryland	throughout	0.60	coarse	steele et al	1994
maize	0.98	USA	north america	non-dryland	throughout	0.61	coarse	steele et al	1994
maize	0.88	USA	north america	non-dryland	throughout	0.56	coarse	steele et al	1994
maize	1.02	USA	north america	non-dryland	throughout	0.69	coarse	steele et al	1994
maize	0.94	USA	north america	non-dryland	throughout	0.57	coarse	steele et al	1994
maize	1.02	USA	north america	non-dryland	throughout	0.74	coarse	steele et al	1994
maize	0.89	USA	north america	non-dryland	throughout	0.54	coarse	steele et al	1994
maize	0.97	USA	north america	non-dryland	throughout	0.86	coarse	steele et al	1994
maize	0.97	USA	north america	non-dryland	throughout	0.77	coarse	steele et al	1994
maize	1.07	USA	north america	non-dryland	throughout	0.57	coarse	steele et al	1994
maize	1.08	USA	north america	non-dryland	throughout	0.85	coarse	steele et al	1994
maize	0.96	USA	north america	non-dryland	throughout	0.67	coarse	steele et al	1994
maize	1.13	USA	north america	non-dryland	throughout	0.61	coarse	steele et al	1994
maize	0.85	USA	north america	non-dryland	throughout	0.38	coarse	steele et al	1994
maize	1.11	USA	north america	non-dryland	throughout	0.70	coarse	steele et al	1994
maize	1.03	USA	north america	non-dryland	throughout	0.65	coarse	steele et al	1994
maize	0.99	USA	north america	dryland	throughout	0.93	medium	yazar et al	1999
maize	0.84	USA	north america	dryland	throughout	0.86	medium	yazar et al	1999
maize	0.78	USA	north america	dryland	throughout	0.79	medium	yazar et al	1999
maize	0.66	USA	north america	dryland	throughout	0.72	medium	yazar et al	1999
maize	0.48	USA	north america	dryland	throughout	0.65	medium	yazar et al	1999
wheat	0.90	mexico	south america	dryland	generative	0.72	n/a	saint pierre et al	2012
wheat	0.87	mexico	south america	dryland	generative	0.60	n/a	saint pierre et al	2012
wheat	0.63	mexico	south america	dryland	throughout	0.45	n/a	saint pierre et al	2012
wheat	1.06	china	asia	dryland	vegetative	n/a	medium	li et al	2009
wheat	1.04	china	asia	dryland	vegetative	n/a	medium	li et al	2009
wheat	0.96	china	asia	dryland	generative	n/a	medium	li et al	2009
wheat	0.86	china	asia	dryland	vegetative	n/a	medium	li et al	2009
wheat	0.87	china	asia	dryland	generative	n/a	medium	li et al	2009
wheat	0.89	china	asia	dryland	throughout	n/a	medium	li et al	2009
maize	0.86	turkey	asia	dryland	throughout	0.61	fine	yazar et al	2009
maize	0.98	turkey	asia	dryland	throughout	0.84	fine	yazar et al	2009
maize	0.85	turkey	asia	dryland	throughout	0.68	fine	yazar et al	2009
maize	0.83	USA	north america	dryland	vegetative	0.60	medium	musick & dusek	1980
maize	0.77	USA	north america	dryland	vegetative	0.40	medium	musick & dusek	1980
maize	0.87	USA	north america	dryland	vegetative	0.60	medium	musick & dusek	1980
maize	0.66	USA	north america	dryland	generative	0.40	medium	musick & dusek	1980
maize	0.83	USA	north america	dryland	generative	0.60	medium	musick & dusek	1980
maize	0.52	USA	north america	dryland	generative	0.40	medium	musick & dusek	1980
maize	0.67	USA	north america	dryland	generative	0.60	medium	musick & dusek	1980
maize	0.88	USA	north america	dryland	generative	0.80	medium	musick & dusek	1980
maize	0.56	USA	north america	dryland	generative	0.60	medium	musick & dusek	1980
maize	0.73	USA	north america	dryland	vegetative	0.60	medium	musick & dusek	1980
maize	0.57	USA	north america	dryland	vegetative	0.40	medium	musick & dusek	1980
maize	0.73	USA	north america	dryland	vegetative	0.60	medium	musick & dusek	1980
maize	0.46	USA	north america	dryland	generative	0.60	medium	musick & dusek	1980
maize	0.10	USA	north america	dryland	generative	0.40	medium	musick & dusek	1980

maize	0.61	USA	north america	dryland	generative	0.80	medium	musick & dusek	1980
maize	0.33	USA	north america	dryland	generative	0.60	medium	musick & dusek	1980
maize	0.06	USA	north america	dryland	generative	0.40	medium	musick & dusek	1980
maize	0.68	USA	north america	dryland	generative	0.80	medium	musick & dusek	1980
maize	0.32	USA	north america	dryland	generative	0.60	medium	musick & dusek	1980
maize	0.70	USA	north america	dryland	generative	0.80	medium	musick & dusek	1980
maize	1.07	USA	north america	dryland	generative	0.83	medium	musick & dusek	1980
maize	1.01	USA	north america	dryland	vegetative	0.83	medium	musick & dusek	1980
maize	0.00	USA	north america	dryland	throughout	0.17	medium	musick & dusek	1980
maize	0.43	USA	north america	dryland	vegetative	0.33	medium	musick & dusek	1980
maize	0.38	USA	north america	dryland	vegetative	0.33	medium	musick & dusek	1980
maize	0.33	USA	north america	dryland	generative	0.33	medium	musick & dusek	1980
maize	0.29	USA	north america	dryland	throughout	0.50	medium	musick & dusek	1980
maize	0.27	USA	north america	dryland	throughout	0.50	medium	musick & dusek	1980
maize	0.08	USA	north america	dryland	generative	0.50	medium	musick & dusek	1980
maize	0.33	USA	north america	dryland	vegetative	0.50	medium	musick & dusek	1980
maize	0.58	USA	north america	dryland	throughout	0.67	medium	musick & dusek	1980
maize	0.77	USA	north america	dryland	generative	0.67	medium	musick & dusek	1980
maize	0.79	USA	north america	dryland	vegetative	0.67	medium	musick & dusek	1980
wheat	1.00	syria	asia	dryland	throughout	0.75	fine	zhang & oweis	1999
wheat	0.66	syria	asia	dryland	throughout	0.50	fine	zhang & oweis	1999
wheat	0.13	syria	asia	dryland	throughout	0.28	fine	zhang & oweis	1999
wheat	1.12	china	asia	dryland	throughout	0.91	medium	zhang et al	2005
wheat	1.38	china	asia	dryland	generative	0.83	medium	zhang et al	2005
wheat	1.36	china	asia	dryland	generative	0.74	medium	zhang et al	2005
wheat	1.42	china	asia	dryland	vegetative	0.84	medium	zhang et al	2005
wheat	1.36	china	asia	dryland	vegetative	0.80	medium	zhang et al	2005
wheat	0.96	china	asia	dryland	throughout	0.92	medium	zhang et al	2005
wheat	1.14	china	asia	dryland	generative	0.85	medium	zhang et al	2005
wheat	1.04	china	asia	dryland	generative	0.77	medium	zhang et al	2005
wheat	1.17	china	asia	dryland	vegetative	0.85	medium	zhang et al	2005
wheat	1.00	china	asia	dryland	vegetative	0.81	medium	zhang et al	2005
wheat	0.55	canada	north america	dryland	throughout	n/a	n/a	duggan et al	2000
maize	0.64	egypt	africa	dryland	throughout	0.95	fine	el-tantawy et al	2007
maize	0.64	egypt	africa	dryland	throughout	0.93	fine	el-tantawy et al	2007
maize	0.89	turkey	asia	dryland	throughout	0.75	coarse	mengu & ozgurel	2008
maize	0.61	turkey	asia	dryland	throughout	0.59	coarse	mengu & ozgurel	2008
maize	0.55	turkey	asia	dryland	throughout	0.43	coarse	mengu & ozgurel	2008
maize	0.35	turkey	asia	dryland	throughout	0.18	coarse	mengu & ozgurel	2008
maize	0.73	turkey	asia	dryland	throughout	0.78	coarse	mengu & ozgurel	2008
maize	0.53	turkey	asia	dryland	throughout	0.64	coarse	mengu & ozgurel	2008
maize	0.40	turkey	asia	dryland	throughout	0.50	coarse	mengu & ozgurel	2008
maize	0.21	turkey	asia	dryland	throughout	0.28	coarse	mengu & ozgurel	2008
maize	0.96	USA	north america	non-dryland	vegetative	0.88	coarse	stegman	1982
maize	0.95	USA	north america	non-dryland	vegetative	0.85	coarse	stegman	1982
maize	0.94	USA	north america	non-dryland	generative	0.81	coarse	stegman	1982
maize	0.92	USA	north america	non-dryland	vegetative	0.72	coarse	stegman	1982
maize	0.74	USA	north america	non-dryland	generative	0.66	coarse	stegman	1982
maize	0.60	USA	north america	non-dryland	generative	0.62	coarse	stegman	1982
maize	0.18	USA	north america	non-dryland	throughout	0.21	coarse	stegman	1982
maize	0.96	USA	north america	non-dryland	throughout	0.76	coarse	stegman	1982
maize	0.89	USA	north america	non-dryland	generative	0.61	coarse	stegman	1982
maize	0.82	USA	north america	non-dryland	generative	0.69	coarse	stegman	1982
maize	0.80	USA	north america	non-dryland	generative	0.54	coarse	stegman	1982
maize	0.69	USA	north america	non-dryland	generative	0.48	coarse	stegman	1982
maize	0.58	USA	north america	non-dryland	generative	0.49	coarse	stegman	1982
maize	0.29	USA	north america	non-dryland	throughout	0.05	coarse	stegman	1982
maize	0.97	USA	north america	non-dryland	vegetative	0.92	medium	stegman	1982
maize	0.92	USA	north america	non-dryland	generative	0.74	medium	stegman	1982
maize	0.87	USA	north america	non-dryland	generative	0.78	medium	stegman	1982
maize	0.73	USA	north america	non-dryland	generative	0.63	medium	stegman	1982
maize	0.68	USA	north america	non-dryland	generative	0.60	medium	stegman	1982
maize	0.66	USA	north america	non-dryland	generative	0.51	medium	stegman	1982
maize	0.30	USA	north america	non-dryland	throughout	0.00	medium	stegman	1982
maize	0.96	USA	north america	non-dryland	throughout	0.84	coarse	stegman	1982
maize	0.92	USA	north america	non-dryland	generative	0.77	coarse	stegman	1982
maize	0.90	USA	north america	non-dryland	vegetative	0.74	coarse	stegman	1982
maize	0.87	USA	north america	non-dryland	generative	0.71	coarse	stegman	1982
maize	0.85	USA	north america	non-dryland	generative	0.56	coarse	stegman	1982
maize	0.78	USA	north america	non-dryland	generative	0.60	coarse	stegman	1982
maize	0.66	USA	north america	non-dryland	throughout	0.23	coarse	stegman	1982
maize	0.99	USA	north america	non-dryland	throughout	0.81	medium	stegman	1982
maize	0.97	USA	north america	non-dryland	vegetative	0.81	medium	stegman	1982
maize	0.93	USA	north america	non-dryland	generative	0.70	medium	stegman	1982
maize	0.93	USA	north america	non-dryland	generative	0.66	medium	stegman	1982
maize	0.92	USA	north america	non-dryland	generative	0.68	medium	stegman	1982
maize	0.89	USA	north america	non-dryland	generative	0.44	medium	stegman	1982
maize	0.68	USA	north america	non-dryland	throughout	0.00	medium	stegman	1982
maize	0.82	brazil	north america	non-dryland	throughout	0.60	fine	soler et al	2007
maize	1.04	USA	north america	dryland	generative	0.65	medium	payero et al	2006b

maize	1.00	USA	north america	dryland	generative	0.67	medium	payero et al	2006b
maize	1.01	USA	north america	dryland	throughout	0.42	medium	payero et al	2006b
maize	1.00	USA	north america	dryland	generative	0.85	medium	payero et al	2006b
maize	0.97	USA	north america	dryland	generative	0.74	medium	payero et al	2006b
maize	0.96	USA	north america	dryland	throughout	0.55	medium	payero et al	2006b
maize	0.94	USA	north america	dryland	generative	0.56	medium	payero et al	2006b
maize	0.89	USA	north america	dryland	generative	0.55	medium	payero et al	2006b
maize	0.70	USA	north america	dryland	throughout	0.40	medium	payero et al	2006b
maize	0.35	USA	north america	dryland	generative	0.43	medium	payero et al	2006b
maize	0.70	USA	north america	dryland	generative	0.45	medium	payero et al	2006b
maize	0.20	USA	north america	dryland	throughout	0.33	medium	payero et al	2006b
maize	1.00	USA	north america	dryland	generative	0.87	medium	payero et al	2006b
maize	1.00	USA	north america	dryland	generative	0.89	medium	payero et al	2006b
maize	0.96	USA	north america	dryland	throughout	0.75	medium	payero et al	2006b
maize	0.29	iran	asia	dryland	throughout	0.63	n/a	golbashy et al	2010
maize	0.93	china	asia	non-dryland	throughout	0.75	medium	ti et al	2010
maize	0.31	china	asia	non-dryland	throughout	0.50	medium	ti et al	2010
maize	0.95	china	asia	non-dryland	throughout	0.75	medium	ti et al	2010
maize	0.19	china	asia	non-dryland	throughout	0.50	medium	ti et al	2010
maize	0.91	turkey	asia	dryland	throughout	0.82	fine	simsek et al	2011b
maize	0.57	turkey	asia	dryland	throughout	0.63	fine	simsek et al	2011b
maize	0.34	turkey	asia	dryland	throughout	0.45	fine	simsek et al	2011b
maize	0.41	spain	europe	dryland	generative	0.77	coarse	farre et al	2000
maize	0.64	spain	europe	dryland	generative	0.76	coarse	farre et al	2000
maize	0.90	spain	europe	dryland	vegetative	0.75	coarse	farre et al	2000
maize	0.82	spain	europe	dryland	generative	0.55	coarse	farre et al	2000
maize	0.64	spain	europe	dryland	generative	0.51	coarse	farre et al	2000
maize	0.94	spain	europe	dryland	generative	0.55	coarse	farre et al	2000
maize	0.25	spain	europe	dryland	throughout	0.31	coarse	farre et al	2000
maize	0.71	spain	europe	dryland	throughout	0.14	coarse	farre et al	2000
maize	0.90	spain	europe	dryland	generative	0.77	coarse	farre et al	2000
maize	0.66	spain	europe	dryland	generative	0.79	coarse	farre et al	2000
maize	0.90	spain	europe	dryland	generative	0.74	coarse	farre et al	2000
maize	0.51	spain	europe	dryland	generative	0.56	coarse	farre et al	2000
maize	0.81	spain	europe	dryland	vegetative	0.53	coarse	farre et al	2000
maize	0.93	spain	europe	dryland	generative	0.50	coarse	farre et al	2000
maize	0.48	spain	europe	dryland	throughout	0.30	coarse	farre et al	2000
maize	0.76	spain	europe	dryland	throughout	0.51	coarse	farre et al	2000
maize	0.74	spain	europe	dryland	throughout	0.82	coarse	farre et al	2000
maize	0.41	spain	europe	dryland	throughout	0.64	coarse	farre et al	2000
maize	0.22	spain	europe	dryland	throughout	0.27	coarse	farre et al	2000
maize	0.07	spain	europe	dryland	throughout	0.27	coarse	farre et al	2000
maize	0.01	spain	europe	dryland	throughout	0.18	coarse	farre et al	2000
maize	0.41	spain	europe	dryland	generative	0.79	medium	farre & faci	2009
maize	0.62	spain	europe	dryland	generative	0.77	medium	farre & faci	2009
maize	0.88	spain	europe	dryland	vegetative	0.77	medium	farre & faci	2009
maize	0.81	spain	europe	dryland	generative	0.58	medium	farre & faci	2009
maize	0.59	spain	europe	dryland	generative	0.54	medium	farre & faci	2009
maize	0.94	spain	europe	dryland	generative	0.58	medium	farre & faci	2009
maize	0.96	spain	europe	dryland	throughout	0.35	medium	farre & faci	2009
maize	0.71	spain	europe	dryland	throughout	0.59	medium	farre & faci	2009
maize	0.87	spain	europe	dryland	generative	0.81	medium	farre & faci	2009
maize	0.60	spain	europe	dryland	generative	0.83	medium	farre & faci	2009
maize	0.87	spain	europe	dryland	vegetative	0.78	medium	farre & faci	2009
maize	0.47	spain	europe	dryland	generative	0.63	medium	farre & faci	2009
maize	0.79	spain	europe	dryland	generative	0.61	medium	farre & faci	2009
maize	0.90	spain	europe	dryland	generative	0.59	medium	farre & faci	2009
maize	0.46	spain	europe	dryland	throughout	0.42	medium	farre & faci	2009
maize	0.73	spain	europe	dryland	throughout	0.60	medium	farre & faci	2009
wheat	0.59	china	asia	dryland	throughout	0.68	medium	kang et al	2002
wheat	0.83	china	asia	dryland	vegetative	0.94	medium	kang et al	2002
wheat	0.73	china	asia	dryland	generative	0.34	medium	kang et al	2002
wheat	0.83	china	asia	dryland	generative	0.62	medium	kang et al	2002
wheat	0.72	china	asia	dryland	throughout	0.50	medium	kang et al	2002
wheat	0.83	china	asia	dryland	generative	0.57	medium	kang et al	2002
wheat	0.81	china	asia	dryland	generative	0.77	medium	kang et al	2002
wheat	0.86	china	asia	dryland	generative	0.85	medium	kang et al	2002
wheat	0.87	china	asia	dryland	generative	0.58	medium	kang et al	2002
wheat	0.87	china	asia	dryland	generative	0.72	medium	kang et al	2002
wheat	0.78	china	asia	dryland	vegetative	0.76	medium	kang et al	2002
wheat	1.02	china	asia	dryland	throughout	0.85	medium	kang et al	2002
wheat	1.06	china	asia	dryland	generative	0.93	medium	kang et al	2002
wheat	1.08	china	asia	dryland	vegetative	0.91	medium	kang et al	2002
wheat	0.42	china	asia	dryland	throughout	0.34	medium	kang et al	2002
wheat	0.76	china	asia	dryland	vegetative	0.57	medium	kang et al	2002
wheat	0.80	china	asia	dryland	generative	0.26	medium	kang et al	2002
wheat	0.93	china	asia	dryland	generative	0.66	medium	kang et al	2002
wheat	0.85	china	asia	dryland	throughout	0.41	medium	kang et al	2002
wheat	0.83	china	asia	dryland	generative	0.45	medium	kang et al	2002
wheat	0.92	china	asia	dryland	generative	0.59	medium	kang et al	2002

wheat	0.96	china	asia	dryland	generative	0.69	medium	kang et al	2002
wheat	0.97	china	asia	dryland	generative	0.53	medium	kang et al	2002
wheat	1.01	china	asia	dryland	generative	0.66	medium	kang et al	2002
wheat	1.01	china	asia	dryland	vegetative	0.74	medium	kang et al	2002
wheat	1.10	china	asia	dryland	throughout	0.74	medium	kang et al	2002
wheat	1.14	china	asia	dryland	generative	0.94	medium	kang et al	2002
wheat	1.17	china	asia	dryland	vegetative	0.96	medium	kang et al	2002
wheat	0.40	china	asia	dryland	throughout	0.68	medium	kang et al	2002
wheat	0.75	china	asia	dryland	vegetative	0.84	medium	kang et al	2002
wheat	0.50	china	asia	dryland	generative	0.29	medium	kang et al	2002
wheat	0.44	china	asia	dryland	generative	0.63	medium	kang et al	2002
wheat	1.00	china	asia	dryland	throughout	0.66	medium	kang et al	2002
wheat	0.50	china	asia	dryland	generative	0.50	medium	kang et al	2002
wheat	0.75	china	asia	dryland	generative	0.72	medium	kang et al	2002
wheat	0.69	china	asia	dryland	generative	0.77	medium	kang et al	2002
wheat	0.76	china	asia	dryland	generative	0.60	medium	kang et al	2002
wheat	0.95	china	asia	dryland	generative	0.75	medium	kang et al	2002
wheat	0.50	china	asia	dryland	vegetative	0.73	medium	kang et al	2002
wheat	1.17	china	asia	dryland	throughout	0.78	medium	kang et al	2002
wheat	1.18	china	asia	dryland	generative	0.97	medium	kang et al	2002
wheat	1.18	china	asia	dryland	vegetative	0.92	medium	kang et al	2002
maize	0.09	mexico	south america	non-dryland	generative	n/a	fine	bolanos & edmeades	1993
maize	0.30	mexico	south america	non-dryland	generative	n/a	fine	edmeades et al	1999
maize	0.00	australia	australia	non-dryland	generative	0.40	medium	carberry et al	1989
maize	0.10	australia	australia	non-dryland	generative	0.44	medium	carberry et al	1989
maize	0.96	australia	australia	non-dryland	generative	0.86	medium	carberry et al	1989
maize	0.73	australia	australia	non-dryland	generative	0.46	medium	carberry et al	1989
maize	0.68	australia	australia	non-dryland	vegetative	0.74	medium	carberry et al	1989
maize	0.22	australia	australia	non-dryland	generative	0.70	medium	carberry et al	1989
maize	0.89	australia	australia	non-dryland	vegetative	0.51	medium	carberry et al	1989
maize	0.52	australia	australia	non-dryland	generative	0.21	medium	carberry et al	1989
maize	0.77	venezuela	south america	non-dryland	vegetative	n/a	medium	sobrado	1990
maize	0.57	venezuela	south america	non-dryland	vegetative	n/a	medium	sobrado	1990
maize	0.88	iran	asia	dryland	vegetative	n/a	fine	ghooshchi et al	2008
maize	0.58	iran	asia	dryland	generative	n/a	fine	ghooshchi et al	2008
maize	0.85	iran	asia	dryland	generative	n/a	fine	ghooshchi et al	2008
maize	1.00	USA	north america	dryland	vegetative	0.89	medium	gordon et al	1995
maize	0.67	USA	north america	dryland	generative	0.77	medium	gordon et al	1995
maize	0.51	USA	north america	dryland	generative	0.66	medium	gordon et al	1995
maize	0.84	USA	north america	dryland	throughout	0.67	medium	gordon et al	1995
maize	1.03	USA	north america	dryland	vegetative	0.85	medium	gordon et al	1995
maize	0.63	USA	north america	dryland	generative	0.55	medium	gordon et al	1995
maize	0.21	USA	north america	dryland	generative	0.43	medium	gordon et al	1995
maize	0.89	USA	north america	dryland	throughout	0.67	medium	gordon et al	1995
maize	1.01	USA	north america	dryland	vegetative	0.92	medium	gordon et al	1995
maize	0.78	USA	north america	dryland	generative	0.85	medium	gordon et al	1995
maize	0.57	USA	north america	dryland	generative	0.77	medium	gordon et al	1995
maize	0.97	USA	north america	dryland	throughout	0.67	medium	gordon et al	1995
maize	1.00	USA	north america	dryland	generative	0.91	medium	gordon et al	1995
maize	0.92	USA	north america	dryland	generative	0.82	medium	gordon et al	1995
maize	0.77	USA	north america	dryland	throughout	0.67	medium	gordon et al	1995
maize	1.00	USA	north america	dryland	generative	0.88	medium	gordon et al	1995
maize	0.98	USA	north america	dryland	generative	0.77	medium	gordon et al	1995
maize	0.97	USA	north america	dryland	throughout	0.67	medium	gordon et al	1995
maize	1.00	USA	north america	dryland	generative	0.87	medium	gordon et al	1995
maize	0.95	USA	north america	dryland	generative	0.75	medium	gordon et al	1995
maize	0.79	USA	north america	dryland	throughout	0.67	medium	gordon et al	1995
maize	1.04	USA	north america	dryland	generative	0.91	medium	gordon et al	1995
maize	1.00	USA	north america	dryland	generative	0.82	medium	gordon et al	1995
maize	0.37	USA	north america	dryland	throughout	0.73	medium	gordon et al	1995
maize	0.94	USA	north america	dryland	generative	0.90	medium	gordon et al	1995
maize	0.89	USA	north america	dryland	generative	0.81	medium	gordon et al	1995
maize	0.67	USA	north america	dryland	throughout	0.71	medium	gordon et al	1995
maize	0.88	USA	north america	dryland	generative	0.90	medium	gordon et al	1995
maize	0.68	USA	north america	dryland	generative	0.79	medium	gordon et al	1995
maize	0.11	USA	north america	dryland	throughout	0.69	medium	gordon et al	1995
maize	0.90	USA	north america	dryland	generative	0.89	medium	gordon et al	1995
maize	0.63	USA	north america	dryland	generative	0.78	medium	gordon et al	1995
maize	0.32	USA	north america	dryland	throughout	0.67	medium	gordon et al	1995
maize	0.95	USA	north america	dryland	generative	0.91	medium	gordon et al	1995
maize	0.90	USA	north america	dryland	generative	0.82	medium	gordon et al	1995
maize	0.28	USA	north america	dryland	throughout	0.73	medium	gordon et al	1995
maize	1.00	USA	north america	dryland	generative	0.91	medium	gordon et al	1995
maize	0.87	USA	north america	dryland	generative	0.82	medium	gordon et al	1995
maize	0.71	USA	north america	dryland	throughout	0.73	medium	gordon et al	1995
maize	0.92	USA	north america	dryland	generative	0.90	medium	gordon et al	1995
maize	0.91	USA	north america	dryland	generative	0.80	medium	gordon et al	1995
maize	0.69	USA	north america	dryland	throughout	0.70	medium	gordon et al	1995
maize	0.96	USA	north america	dryland	generative	0.88	medium	gordon et al	1995
maize	0.81	USA	north america	dryland	generative	0.75	medium	gordon et al	1995

maize	0.43	USA	north america	dryland	throughout	0.63	medium	gordon et al	1995
maize	0.97	USA	north america	dryland	generative	0.90	medium	gordon et al	1995
maize	0.88	USA	north america	dryland	generative	0.80	medium	gordon et al	1995
maize	0.30	USA	north america	dryland	throughout	0.70	medium	gordon et al	1995
maize	0.98	USA	north america	dryland	generative	0.91	medium	gordon et al	1995
maize	0.88	USA	north america	dryland	generative	0.81	medium	gordon et al	1995
maize	0.31	USA	north america	dryland	throughout	0.72	medium	gordon et al	1995
maize	0.95	USA	north america	dryland	generative	0.87	medium	gordon et al	1995
maize	0.80	USA	north america	dryland	generative	0.74	medium	gordon et al	1995
maize	0.02	USA	north america	dryland	throughout	0.61	medium	gordon et al	1995
maize	1.02	USA	north america	non-dryland	throughout	0.87	coarse	vories et al	2009
maize	0.75	USA	north america	non-dryland	throughout	0.57	coarse	vories et al	2009
maize	1.05	USA	north america	non-dryland	throughout	0.89	coarse	vories et al	2009
maize	0.97	USA	north america	non-dryland	throughout	0.68	coarse	vories et al	2009
maize	0.99	USA	north america	non-dryland	throughout	0.93	coarse	vories et al	2009
maize	0.94	USA	north america	non-dryland	throughout	0.77	coarse	vories et al	2009
maize	0.99	USA	north america	dryland	throughout	0.94	medium	howell et al	1995
maize	0.84	USA	north america	dryland	throughout	0.88	medium	howell et al	1995
maize	0.78	USA	north america	dryland	throughout	0.85	medium	howell et al	1995
maize	0.66	USA	north america	dryland	throughout	0.75	medium	howell et al	1995
maize	0.48	USA	north america	dryland	throughout	0.68	medium	howell et al	1995
maize	0.96	USA	north america	dryland	throughout	0.87	medium	howell et al	1995
maize	0.83	USA	north america	dryland	throughout	0.88	medium	howell et al	1995
maize	0.70	USA	north america	dryland	throughout	0.92	medium	howell et al	1995
maize	0.50	USA	north america	dryland	throughout	0.75	medium	howell et al	1995
maize	0.26	USA	north america	dryland	throughout	0.59	medium	howell et al	1995
maize	0.89	USA	north america	dryland	throughout	0.33	medium	tolk et al	1998
maize	0.89	USA	north america	dryland	throughout	0.33	medium	tolk et al	1998
maize	0.84	USA	north america	dryland	throughout	0.33	coarse	tolk et al	1998
maize	0.90	USA	north america	dryland	throughout	0.63	medium	tolk et al	1998
maize	0.97	USA	north america	dryland	throughout	0.63	medium	tolk et al	1998
maize	0.78	USA	north america	dryland	throughout	0.63	coarse	tolk et al	1998
maize	0.98	USA	north america	dryland	throughout	0.75	medium	tolk et al	1998
maize	0.85	USA	north america	dryland	throughout	0.49	medium	tolk et al	1998
maize	0.62	USA	north america	dryland	throughout	0.23	medium	tolk et al	1998
maize	0.95	USA	north america	dryland	throughout	0.74	medium	tolk et al	1998
maize	0.93	USA	north america	dryland	throughout	0.49	medium	tolk et al	1998
maize	0.77	USA	north america	dryland	throughout	0.23	medium	tolk et al	1998
maize	0.97	USA	north america	dryland	throughout	0.74	coarse	tolk et al	1998
maize	0.91	USA	north america	dryland	throughout	0.49	coarse	tolk et al	1998
maize	0.73	USA	north america	dryland	throughout	0.23	coarse	tolk et al	1998
maize	0.82	USA	north america	dryland	throughout	n/a	medium	mason et al	2008
wheat	0.58	mexico	south america	dryland	generative	0.33	coarse	monneveux et al	2005
wheat	0.63	mexico	south america	dryland	vegetative	0.44	coarse	monneveux et al	2005
wheat	0.49	mexico	south america	dryland	throughout	0.11	coarse	monneveux et al	2005
wheat	0.64	mexico	south america	dryland	generative	0.33	coarse	monneveux et al	2005
wheat	0.69	mexico	south america	dryland	vegetative	0.44	coarse	monneveux et al	2005
wheat	0.62	mexico	south america	dryland	throughout	0.11	coarse	monneveux et al	2005
maize	0.95	iran	asia	dryland	generative	n/a	n/a	gholamin & khayatnezhad	2011
maize	0.66	turkey	asia	dryland	throughout	0.98	medium	kara & biber	2008
maize	0.74	turkey	asia	dryland	throughout	0.99	medium	kara & biber	2008
maize	0.27	turkey	asia	dryland	throughout	0.68	medium	kara & biber	2008
wheat	0.69	iran	asia	dryland	throughout	n/a	n/a	naserian et al	2007
wheat	0.57	iran	asia	dryland	generative	n/a	n/a	dadbakhsh et al	2011
wheat	0.89	iran	asia	dryland	generative	n/a	coarse	khamssi	2011
wheat	0.85	iran	asia	dryland	generative	n/a	medium	eskandari & kazemi	2010
maize	0.92	spain	europe	dryland	generative	0.51	coarse	aguilar et al	2007
maize	0.97	spain	europe	dryland	generative	0.64	coarse	aguilar et al	2007
maize	0.62	spain	europe	dryland	generative	0.66	coarse	aguilar et al	2007
maize	0.73	spain	europe	dryland	generative	0.62	coarse	aguilar et al	2007
maize	0.42	ethiopia	africa	dryland	throughout	0.25	medium	Mengiste & Tilahun	2009
maize	0.97	ethiopia	africa	dryland	vegetative	0.92	medium	Mengiste & Tilahun	2009
maize	0.94	ethiopia	africa	dryland	vegetative	0.84	medium	Mengiste & Tilahun	2009
maize	0.79	ethiopia	africa	dryland	generative	0.60	medium	Mengiste & Tilahun	2009
maize	0.97	ethiopia	africa	dryland	generative	0.89	medium	Mengiste & Tilahun	2009
maize	0.97	ethiopia	africa	dryland	vegetative	0.95	medium	Mengiste & Tilahun	2009
maize	0.96	ethiopia	africa	dryland	vegetative	0.90	medium	Mengiste & Tilahun	2009
maize	0.86	ethiopia	africa	dryland	generative	0.73	medium	Mengiste & Tilahun	2009
maize	0.96	ethiopia	africa	dryland	generative	0.93	medium	Mengiste & Tilahun	2009
maize	0.77	USA	north america	dryland	throughout	0.46	medium	van donk et al	2012
maize	0.84	USA	north america	dryland	throughout	0.58	medium	van donk et al	2012
maize	0.95	USA	north america	dryland	throughout	0.79	medium	van donk et al	2012
maize	0.95	USA	north america	dryland	generative	0.75	medium	van donk et al	2012
maize	0.94	USA	north america	dryland	generative	0.75	medium	van donk et al	2012
maize	0.97	USA	north america	dryland	generative	0.86	medium	van donk et al	2012
maize	1.00	USA	north america	dryland	generative	0.86	medium	van donk et al	2012
maize	0.75	USA	north america	dryland	throughout	0.56	medium	van donk et al	2012
maize	0.95	USA	north america	dryland	throughout	0.71	medium	van donk et al	2012
maize	0.99	USA	north america	dryland	throughout	0.86	medium	van donk et al	2012
maize	1.00	USA	north america	dryland	generative	0.83	medium	van donk et al	2012

maize	1.01	USA	north america	dryland	generative	0.85	medium	van donk et al	2012
maize	1.00	USA	north america	dryland	generative	0.89	medium	van donk et al	2012
maize	0.98	USA	north america	dryland	generative	0.95	medium	van donk et al	2012
maize	1.02	USA	north america	dryland	throughout	0.58	medium	van donk et al	2012
maize	0.98	USA	north america	dryland	throughout	0.59	medium	van donk et al	2012
maize	1.00	USA	north america	dryland	throughout	0.80	medium	van donk et al	2012
maize	0.98	USA	north america	dryland	generative	0.75	medium	van donk et al	2012
maize	1.05	USA	north america	dryland	generative	0.59	medium	van donk et al	2012
maize	0.99	USA	north america	dryland	generative	0.94	medium	van donk et al	2012
wheat	0.62	denmark	europe	non-dryland	throughout	n/a	coarse	imtiyaz et al	1982
wheat	0.52	denmark	europe	non-dryland	throughout	0.50	coarse	talukder et al; mogensen & talukder	1987
wheat	1.00	denmark	europe	non-dryland	vegetative	0.83	coarse	talukder et al; mogensen & talukder	1987
wheat	0.88	denmark	europe	non-dryland	generative	0.75	coarse	talukder et al; mogensen & talukder	1987
wheat	0.72	denmark	europe	non-dryland	generative	0.50	coarse	talukder et al; mogensen & talukder	1987
wheat	1.06	india	asia	dryland	throughout	0.22	coarse	aggarwal & sinha	1987
wheat	0.23	india	asia	dryland	throughout	0.31	coarse	aggarwal & sinha	1987
wheat	0.39	india	asia	dryland	throughout	0.40	coarse	aggarwal & sinha	1987
wheat	0.33	india	asia	dryland	throughout	0.35	coarse	aggarwal & sinha	1987
wheat	0.43	india	asia	dryland	throughout	0.44	coarse	aggarwal & sinha	1987
wheat	0.60	india	asia	dryland	throughout	0.53	coarse	aggarwal & sinha	1987
maize	0.71	USA	north america	dryland	generative	0.70	medium	jama & ottman	1993
maize	0.62	USA	north america	dryland	generative	0.69	medium	jama & ottman	1993
maize	0.58	USA	north america	dryland	generative	0.66	medium	jama & ottman	1993
maize	0.56	USA	north america	dryland	generative	0.78	medium	jama & ottman	1993
maize	0.62	USA	north america	dryland	generative	0.77	medium	jama & ottman	1993
maize	0.67	USA	north america	dryland	generative	0.76	medium	jama & ottman	1993
wheat	0.88	iran	asia	dryland	throughout	0.86	medium	fardad & pessarakli	1995
wheat	0.86	iran	asia	dryland	throughout	0.73	medium	fardad & pessarakli	1995
wheat	0.67	iran	asia	dryland	throughout	0.60	medium	fardad & pessarakli	1995
wheat	0.55	iran	asia	dryland	throughout	0.46	medium	fardad & pessarakli	1995
wheat	0.34	iran	asia	dryland	throughout	0.33	medium	fardad & pessarakli	1995
wheat	0.95	iran	asia	dryland	throughout	0.87	medium	fardad & pessarakli	1995
wheat	0.79	iran	asia	dryland	throughout	0.74	medium	fardad & pessarakli	1995
wheat	0.67	iran	asia	dryland	throughout	0.62	medium	fardad & pessarakli	1995
wheat	0.58	iran	asia	dryland	throughout	0.49	medium	fardad & pessarakli	1995
wheat	0.40	iran	asia	dryland	throughout	0.36	medium	fardad & pessarakli	1995
maize	0.37	brazil	south america	non-dryland	generative	n/a	medium	de souza et al	2013
maize	0.70	brazil	south america	non-dryland	generative	n/a	medium	de souza et al	2013
maize	0.42	nigeria	africa	non-dryland	generative	n/a	fine	menkir et al	2009
maize	1.02	USA	north america	non-dryland	throughout	0.83	coarse	powell & wright	1993
maize	0.85	USA	north america	non-dryland	throughout	0.78	coarse	powell & wright	1993
maize	0.75	USA	north america	non-dryland	throughout	0.64	coarse	powell & wright	1993
maize	0.28	USA	north america	non-dryland	throughout	0.35	coarse	powell & wright	1993
maize	1.01	USA	north america	non-dryland	throughout	0.68	coarse	powell & wright	1993
maize	0.72	USA	north america	non-dryland	throughout	0.44	coarse	powell & wright	1993
maize	1.01	USA	north america	non-dryland	throughout	0.69	coarse	powell & wright	1993
maize	1.07	USA	north america	non-dryland	throughout	0.34	coarse	powell & wright	1993
maize	0.99	USA	north america	non-dryland	throughout	0.64	coarse	powell & wright	1993
maize	0.61	USA	north america	non-dryland	throughout	0.39	coarse	powell & wright	1993
